# Design, Synthesis, and Anti-Cancer Evaluation of Novel Water-Soluble Copper(I) Complexes Bearing Terpyridine and PTA Ligands

**DOI:** 10.3390/molecules29050945

**Published:** 2024-02-21

**Authors:** Piotr Smoleński, Urszula Śliwińska-Hill, Anna Kwiecień, Joanna Wolińska, Dominik Poradowski

**Affiliations:** 1 Faculty of Chemistry, University of Wrocław, F. Joliot-Curie 14, 50-383 Wrocław, Poland; 2 Department of Basic Chemical Sciences, Faculty of Pharmacy, Wrocław Medical University, Borowska 211a, 50-556 Wrocław, Poland; urszula.sliwinska-hill@umw.edu.pl (U.Ś.-H.);; 3 Department of Biostructure and Animal Physiology, Faculty of Veterinary Medicine, Wrocław University of Environmental and Life Sciences, Kożuchowska 1, 51-631 Wrocław, Poland; 118689@student.upwr.edu.pl (J.W.);

**Keywords:** water-soluble copper(I) complexes, NMR test, MTT test, cytotoxicity, spectroscopy

## Abstract

This study presents a simple and energy-efficient self-assembly LAG synthetic method for novel water-soluble copper(I) complexes [Cu(terpy)(PTA)][PF_6_] (**1**) and [Cu(terpy)(PTA)_2_][PF_6_] (**2**). They were characterized by FT-IR, ^1^H, and ^31^P{^1^H} NMR spectroscopy, elemental analysis, and single-crystal/powder X-ray diffraction (for **2**). The X-ray analysis of compound **2** indicates a bidentate coordination mode of terpyridine to the metal center. Variable-temperature NMR tests indicate dynamic properties for terpyridine in the case of both compounds, as well as for the PTA ligands in the case of **2**. Additionally, compounds **1** and **2** exhibit interesting cytotoxic activity, which was tested on normal human dermal fibroblasts (NHDFs), human lung carcinoma (A549), human breast adenocarcinoma (MCF-7), and human cervix carcinoma (HeLa) established cell lines. In comparison to the other tested compounds, complexes **1** and **2** seem to have significantly lower IC_50_ values against cancer cells (A549, HeLa, MCF-7), indicating their potential as prospective anticancer agents. Moreover, both compounds show no significant toxicity towards normal skin cells (NHDFs), suggesting a certain selectivity in their action on cancer cells. Cisplatin as a reference compound also exhibited considerable cytotoxicity against cancer cells but with a low level of selectivity, which could lead to unwanted effects on normal cells. Remarkably, compounds **1** and **2** exhibit up to 30 times the cytotoxic activity of cisplatin, with a six-fold lower toxicity to normal cells. They also interact strongly with human serum albumin, suggesting potential therapeutic applications. Overall, these compounds hold significant promise as potential chemotherapeutic agents.

## 1. Introduction

Cancer is a serious disease contributing to the premature death of many people around the world. It is predicted that over the next 50 years, the number of cancer cases may increase due to demographic changes [1]. Therefore, it is essential to constantly search for new and more effective drugs in the fight against this disease. In the course of this, many coordination compounds of various transition metals have been studied, and among them, copper complexes occupy a prominent place. First of all, copper is essential for the proper functioning of many organisms [2]. It takes part in cellular respiration, iron oxidation, the formation of pigments, and biosynthesis of neurotransmitters as well as in the development of the central nervous system, the antioxidant defense of the body, and the formation of connective tissue. In organisms, copper is present both in the Cu(I) and Cu(II) forms, which are approximately 90% bound by ceruloplasmin [2]. Secondly, many copper compounds have been successfully tested as potential anti-cancer agents [2,3,4,5,6,7].

In particular, the compounds of copper with terpyridine (terpy = 2,2′:6′,2″-terpyridine) have been studied for their biological activity, although the ligand has also been successfully used as a photosensitizer in many coordination compounds reported in the past [8]. Terpyridine–transition metal complexes are used in photochemistry for the design of luminescent devices or sensitizers in clinical chemistry and biochemistry for the calorimetric determination of metals, DNA binding, and anti-cancer research [8].

However, in the case of copper–terpyridine complexes, most of the reported compounds are copper(II), in contrast to copper(I) complexes, of which only a few examples have been structurally characterized and reported [9,10,11,12]. Previously described were four- and five-coordinated copper(I) complexes with terpyridine and its derivatives and tertiary phosphines such as triphenylphosphine [10,11,12] and bis(2-(diphenylphosphine)-phenyl)ether [9]. The terpyridine in these compounds coordinates in the bi- [9,12] and tridentate [9,10,11] modes to the Cu(I) metal center. However, phosphorus(III) ligands used for syntheses do not induce a polar character and the hydrosolubility of the obtained compounds. Moreover, the authors stated the compounds’ low stability, especially in solutions. Therefore, a good alternative to conventional phosphines in this type of compound might be the air-stable and water-soluble aminophosphine 1,3,5-triaza-7-phosphaadamantane (PTA). In the past, the coordination chemistry of PTA ligands has seen a pronounced development justified by the search for water-soluble and stable transition metal complexes [13,14,15,16,17,18,19,20,21,22,23]. In particular, some of us have reported several stable water-soluble copper– and silver–PTA-cage coordination networks as well as discrete complexes [24,25,26,27,28,29,30,31,32]. Representative compounds have also been tested successfully for their cytotoxic, antibacterial, and antifungal activity, as well as their DNA binding properties [24,25,26,27,28,29,30,31,33,34].

Recently, we have described the synthesis, full characterization, and significant cytotoxic activity of a heteroleptic, water-soluble silver complex with terpyridine and the PTA ligand [Ag(terpy)(PTA)][NO_3_] [30]. By further extending these studies from terpyridine ligands, we now report the LAG route generation and full characterization of the new copper(I) complexes [Cu(terpy)(PTA)][PF_6_] (1) and [Cu(terpy)(PTA)_2_][PF_6_] (2). These complexes were evaluated in aqua media for their cytotoxic activities on the normal human dermal fibroblast (NHDF) cell line and their antitumor activity using the human lung carcinoma (A549), epithelioid cervix carcinoma (HeLa), and human breast adenocarcinoma (MCF-7) cell lines. In addition, the interactions between the representative complexes and apo-transferrin were investigated by circular dichroism (CD), fluorescence, and UV-Vis spectroscopy.

## 2. Results and Discussion

### 2.1. Synthesis and Characterization

**Scheme 1:**
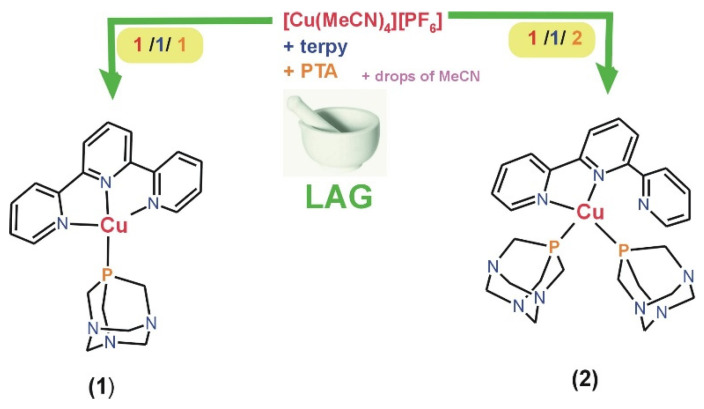
Schematic representation of the mechanochemical synthesis of **1** and **2**.

**Figure 1:**
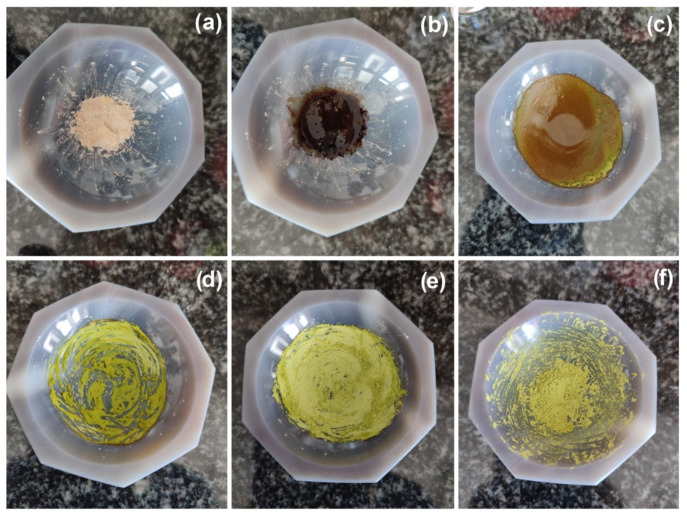
Monitoring of the LAG synthesis of **1** (images obtained under day-light): (**a**) a dry mixture of [Cu(MeCN)_4_][PF_6_] with terpy and PTA in 1:1:1 molar ratio; (**b**) the reaction mixture with 80 µL of MeCN; (**c**–**f**) the reaction mixture after 30, 60, 90, and 120 s grinding, respectively.

Sustainable synthetic approaches to producing functional coordination compounds hold paramount significance for both the environment and the economy. Therefore, using terpyridine, PTA, and copper(I) precursor, herein, we synthesized, mechanochemically, using liquid-assisted grinding (LAG) two copper(I) coordination compounds, [Cu(terpy)(PTA)][PF_6_] (**1**) and [Cu(terpy)(PTA)_2_][PF_6_] (**2**). The synthesis process employs the straightforward application of a pestle and mortar with an *ƞ* range of 0.82–0.90 µL·mg^−1^. A stoichiometric two-minute LAG reaction (ratio 1:1:1 and 1:1:2) of [Cu(MeCN)_4_][PF_6_], terpyridine, and PTA ligands give, with high yield, [Cu(terpy)(PTA)][PF_6_] (**1**) and [Cu(terpy)(PTA)_2_][PF_6_] (**2**), respectively (Scheme 1 and Figure 1). Furthermore, as detailed in this context, the LAG synthetic procedure proves to be rapid, uncomplicated, and energy-efficient, thereby reducing its environmental footprint. Alternatively, compounds **1**–**2** could be prepared through conventional synthesis in acetonitrile as a solvent. However, this method yields significantly less (see Section 3).

The products **1**–**2** were isolated as air- and moisture-stable yellow microcrystalline solids, and characterized by FT-IR, ^1^H, and ^31^P{^1^H} NMR spectroscopy, elemental analyses, and single-crystal (for **2**)/powder X-ray diffraction (for **1** and **2**). In addition, a diffractogram generated for compound **2** using single-crystal X-ray data corresponds well with the experimental PXRD patterns shown in Figure S1. This confirms that the product obtained after both mechanochemical and wet syntheses is pure and homogeneous. However, the PXRD patterns of compound **1** suggest different molecular properties as compared to complex **2**, as seen in Figure S2. A significant feature of **1**–**2** concerns their hydrosolubility, with the *S*_25°C_ values for **1** and **2** being 5 and 23 mg∙mL^−1^, respectively. In addition, these compounds are soluble in DMSO, MeCN, and acetone and are slightly soluble in methanol, ethanol, and THF. The compounds are stable in solutions under an inert atmosphere, and when exposed to air, they slowly decompose, changing color from yellow to greenish-yellow after one day for **1** and one week for **2**, respectively. The ^31^P{^1^H} NMR spectra of **1** and **2** in acetone-*d*_6_, which were prepared in air, showed the progressive decrease of the original ^31^P{^1^H} resonance of complexes **1** and **2**, with the concomitant increase of the free PTA = O singlet, the process being accomplished in one day and one week, for **1** and **2**, respectively. On the other hand, in the sample of the aqueous solution of **1** that was left in the air for a month, the presence of [Cu(terpy)(PTA)_2_][PF_6_] (**2**), [Cu(terpy)_2_][PF_6_]_2_ [35], [Cu(PTA)_4_][PF_6_] [36,37], and PTA = O [38] was detected using X-ray structural analysis. This indicates the significantly lower stability of compound **1** in comparison to **2** in solutions, especially considering that one of the decomposition/disproportionation products of compound **1** is, among others, compound **2**.

**Figure 2:**
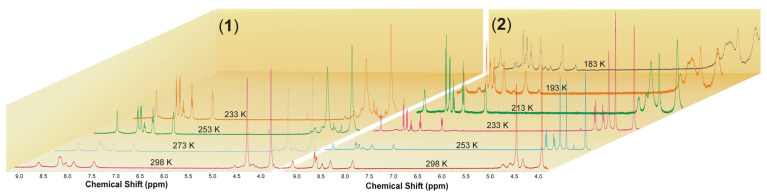
Variable temperature ^1^H NMR spectra of **1** and **2** in acetone-*d*_6_.

The ^1^H NMR spectra of **1** and **2** in acetone-*d*_6_ at room temperature exhibit a set of broad signals expected for the aromatic protons of terpyridine along with the methylene protons of PTA ligands (Figure 2 and Figures S7–S20). Broad signals are observed due to the average effect of a dynamic process for terpyridine in the case of compound **1** and for both terpyridine and PTA in the case of compound **2**. At lower temperatures, a split of the terpyridine signals can be observed in the case of [Cu(terpy)(PTA)][PF_6_] (**1**), giving a typical set of resonances for a symmetrically coordinated ligand, with signals from PTA undergoing only a slight broadening as the temperature decreases. At lower temperatures, the ^1^H NMR spectra of [Cu(terpy)(PTA)_2_][PF_6_] (**2**) exhibit a split of the terpyridine signals as well as a conversion to a double set of resonances in a 1:1 ratio for the PTA ligands. The first set consists of two singlets found at 3.85 and 4.24 ppm, while the second includes a singlet at 4.37 ppm and an AB spin system resonance centered at 4.57 ppm. These can be assigned to the methylene bridges that connect the phosphorus and nitrogen atoms (P-CH_2_-N) and nitrogen atoms themselves (N-CH_2_-N), respectively.

**Figure 3:**
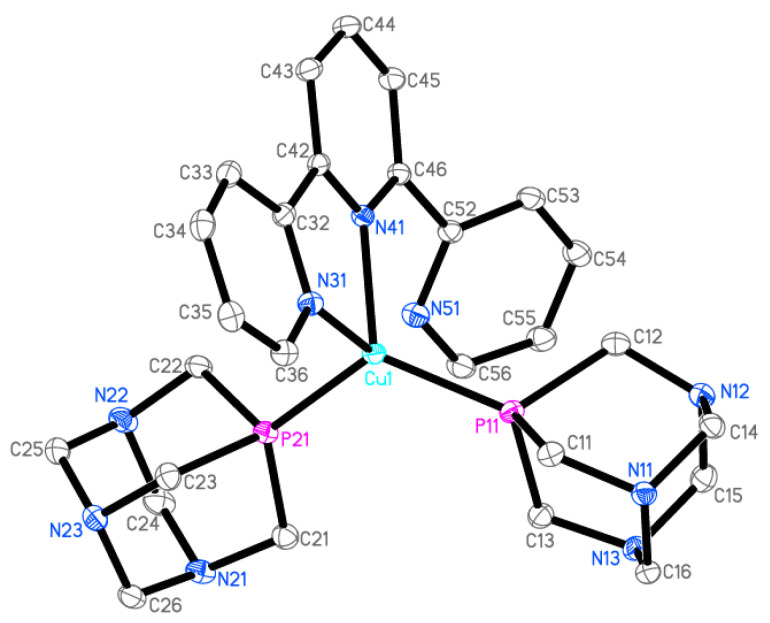
Ellipsoid diagram (drawn at 30% probability level) of **2** cation, hydrogen atoms, and [PF_6_]^−^ anion omitted for clarity.

It is essential to note that when the temperature ranges from 253 K to 233 K, the ^1^H NMR spectra of **2** in the aromatic region also show distinctive and well-separated resonances that correspond to the terpyridine ligand. This observation suggests the possibility of the terpy domain functioning as a tridentate ligand, leading to a five-coordinate copper(I) center. Alternatively, it could indicate that the terpy acts as a bidentate ligand, undergoing dynamic behavior on the NMR timescale with a low energy barrier to the process. As previously stated, at low temperatures, we can detect the ^1^H NMR spectrum signals from two distinct PTA molecules which may be a result of terpyridine’s asymmetric coordination to the metal center. This can affect the strengths of the Cu-P1 and Cu-P2 bonds (Figure 3). However, we can see from the structural data in the solid state that the bond lengths between the copper atom and the phosphorus atoms of the PTA molecules have only slight differences, as shown in Table 1 and Table 2. Below the temperature of 233 K, in the case of compound **2**, the broadening of the signals originating from terpyridine and PTA is noticeable. Furthermore, when the temperature decreases to 193 K, other signals in the aromatic region became visible. Upon further cooling to 183 K, close to the solvent’s freezing point, a new set of resonances from terpyridine with different coordination modes appeared. These resonances were centered at 8.77, 8.72, 8.56, 8.15, 8.04, and 7.52 ppm and had lower intensities compared to the original signals, appearing at an approximate ratio of 1:8 (Figure S19). At low temperatures, the ^31^P{^1^H} NMR spectra of **1** and **2** show broad singlets at *δ* −93.3 and −93.5 from the coordinated PTA molecule, as well as resonances from the [PF6]^−^ counterion, centered at *δ* −143.7 (septet, *J*_PF_ = 710.2 Hz) and −143.8 (septet, *J*_PF_ = 708.7 Hz), respectively (Figures S12 and S20). 

The infrared (IR) spectra of **1**–**2** display certain vibrations of coordinated terpyridine and PTA ligands (Figures S3–S6). The absorption due to several *ν*(CH) bands are detected in the range of 2954–2816 cm^−1^, while some bands in the range of 1600–550 cm^−1^ are associated with *ν*(C-X) (X = C, N, P) vibrations [39]. The IR spectra also indicate the characteristic strong and broad band centered at 843–840 cm^−1^, which belongs to the PF_6_^−^ anion.

Preliminary tests of the luminescent properties of the obtained compounds were also carried out. However, it has been established that complexes **1**–**2** are poorly emissive in the solid state/solutions at room and low temperatures.

### 2.2. Crystal Structure of ***2***

**Figure 4:**
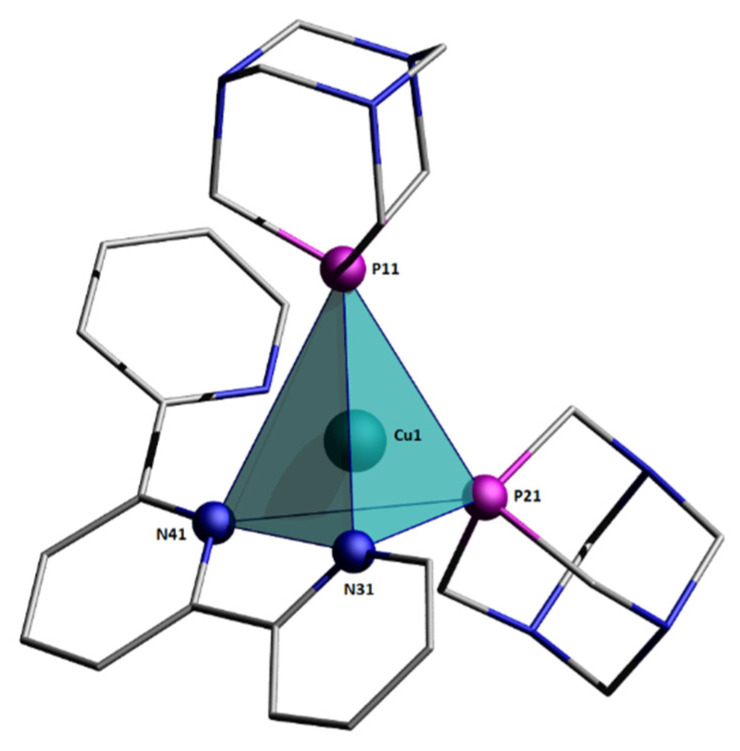
Coordination sphere around Cu(I) center.

The crystallographic data and selected geometrical parameters for [Cu(terpy)(PTA)_2_][PF_6_] (**2**) are presented in Table 1 and Table 2, respectively. The Cu(I) coordination sphere consists of four donor atoms (Figure 3): two phosphorus atoms from two PTA molecules, and two nitrogen atoms from terpyridine. The terpy molecule possesses three nitrogen atoms; thus, it can coordinate as a tridentate ligand. However, in the crystal structure of [Cu(terpy)(PTA)_2_][PF_6_], only two of these nitrogen atoms (N31 and N41) form coordinate bonds with the copper cation. The interatomic distance between the Cu1 and N51 atoms (2.9581(17) Å) is larger than the sum of the van der Waals radii and thus cannot be considered as a coordination bond. The N51 nitrogen atom is involved in an intramolecular hydrogen bond as an acceptor. The values of the bond lengths and valence angles suggest a highly distorted tetrahedral environment (Figure 4). Quadratic elongation and angle variance, parameters describing the distortion in a coordination polyhedral, [40,41] are 1.071 and 229.27 [deg^2^], respectively. Other indices for geometrical shape in four-coordinate systems are the tetrahedral character (THC_DA_) parameter, [42,43] four-coordinate geometric (FCGP), [44] and τ_4_ parameters [45]. Table 3 contains the values of these indices calculated for the metal coordination sphere in the [Cu(terpy)(PTA)_2_]^+^ cation as well as for the ideal geometrical shape of the tetrahedron and trigonal pyramid. 

The parameters THC_DA_ and τ_4_ calculated for [Cu(terpy)(PTA)_2_]^+^ are close to those of the ideal trigonal pyramid, because in equations, only the bond angle values are considered (all six for THC_DA_, Equation (1); and the two largest for τ_4_, Equation (2)) [43,45].

(1) $${THC}_{DA}=\left(1-\frac{\sum_{n=1}^{6}\left|109.5{}^{\circ}-\theta_{n}\right|}{90{}^{\circ}}\right)\times 100$$

(2) $$\tau_{4}=\frac{360{}^{\circ}-(\alpha +\beta )}{141{}^{\circ}}$$

where θ_n_—bond angle, α and β—two largest angles. 

For an ideal trigonal pyramid with *C*_3*V*_ symmetry, the values of the bond angles are θ_1-3_ = 120°, θ_4±6_ = 90°; thus, α = β = 120°. In the coordination polyhedron of the [Cu(terpy)(PTA)_2_]^+^ cation, also, three bond angles are close to 120° (Table 2). However, after a visual inspection of the structure, the coordination sphere around the Cu(I) center should be described as a distorted tetrahedron. None of the three donor atoms are coplanar with the copper atom. In the ideal trigonal pyramid, the bond angles of 120° are between the central metal ion and three different donor atoms laying on the same plane. In the analyzed crystal structure, the three largest bond angles close to 120° involve one donor atom P11 and the central Cu atom is situated inside the coordination polyhedron. 

**Figure 5:**
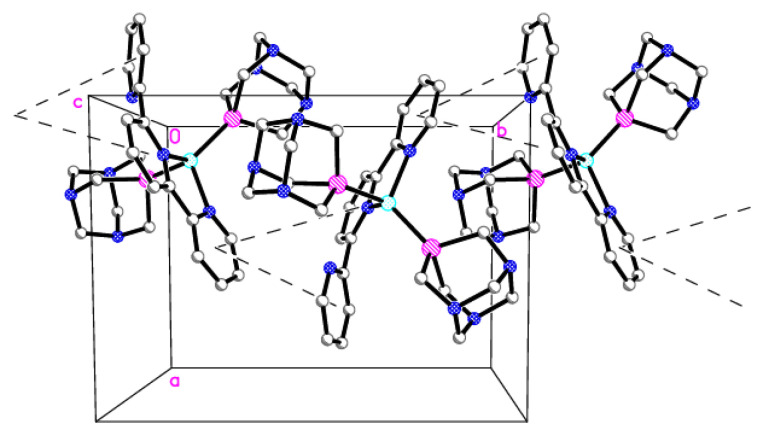
The 1D chain motif of cations of **2** connected by stacking interactions (dashed lines; *a*, *b*, *c*—lattice parameters, 0—origin of the coordinate system). Details: H atoms are omitted, C (gray), N (blue), P (magenta), Cu (cyan).

In the crystal structure is observed a 1D chain motif parallel to the crystallographic axis b (Figure 5). This chain is built of cation moieties interconnected by stacking interactions between the aromatic rings of the terpyridine fragments and further stabilized by a C-H···π type interaction (Tables S1 and S2). The three-dimensional network of noncovalent C-H···N and C-H···F type interaction stabilizes the whole crystal structure (Table S3).

### 2.3. Cytotoxic Activity

Due to the good aqua solubility of compounds **1** and **2**, their cytotoxic activity was tested on normal human dermal fibroblasts (NHDFs), human lung carcinoma (A549), human breast adenocarcinoma (MCF-7) and human cervix carcinoma (HeLa). In in vitro studies, the fibroblast or lymphoblast cell line are most often used as a reference line for normal body cells, which is why we decided to use the NHDF line, like some other authors [46,47,48,49,50,51], in combination with the mentioned cancer lines. Other fibroblast lines can also be used as normal cell lines [52,53,54]. In addition, the reference compounds free terpyridine and PTA, as well as copper(I) precursor [Cu(MeCN)_4_][PF_6_], were also tested. Table 4 presents the IC_50_, which indicates the half-maximal inhibitory concentration. Based on the results obtained, it was found that PTA did not show any signs of toxicity towards the tested cell lines. This was evident as the IC_50_ concentration for each cell line was between 100 and 200 μM/mL. However, the free terpyridine ligand exhibited strong cytotoxicity towards both normal (NHDFs) and cancerous cell lines (A549, MCF-7, HeLa), while the Cu(I) precursor showed intermediate cytotoxicity between the terpyridine and PTA ligands. Various cell lines were used to evaluate the impact of compounds **1** and **2** on the growth of normal and cancerous cells. The results showed that both compounds did not exhibit significant toxicity towards the normal skin cells (NHDF cell line), with IC_50_ values between 100 and 200 μM/mL. However, in the A549 cell line, **1** displayed very strong cytotoxicity, while **2** exhibited moderate cytotoxicity towards lung cancer cells. For cervical cancer cells (HeLa cell line), both compounds showed strong or moderate cytotoxicity, with low IC50 values of 0.31 ± 0.61 and 5.09 ± 0.79, respectively. In the MCF-7 cell line, compound **1** showed significant cytotoxicity towards breast cancer cells, with an IC_50_ value of 1.29 ± 2.47. Meanwhile, **2** exhibited strong cytotoxicity towards breast cancer cells, with an IC_50_ value of 0.23 ± 1.39.

In summary, compound **1** appears to be more effective against cancer cells compared to **2**. Both compounds show no significant toxicity towards normal skin cells (NHDFs), suggesting a certain selectivity in their action on cancer cells. In comparison to the other tested compounds, complexes **1** and **2** seem to have significantly lower IC_50_ values against cancer cells (A549, HeLa, MCF-7), indicating their potential as prospective anticancer agents. Cisplatin also exhibits considerable cytotoxicity against cancer cells but with a low level of selectivity, which could lead to unwanted effects on normal cells. An interesting comparison arises between the investigated compound **1** and a previously published [Ag(terpy)(PTA)][NO_3_], which was described as more selective and active against cancer cell lines compared to the well-known cisplatin [30]. However, the research outlined in this study demonstrated that substituting silver(I) with copper(I) ions resulted in a significant increase not only in the activity against selected cancer cell lines but also in the selectivity towards both healthy and diseased cells. Indeed, compound **1** is considerably more reactive compared to compound **2**, as well as the analogous silver compound. This is evident in its slightly faster decomposition in an aqueous solution when exposed to air, as compared to the other compounds discussed.

### 2.4. Apo-Transferrin Interactions

**Figure 6:**
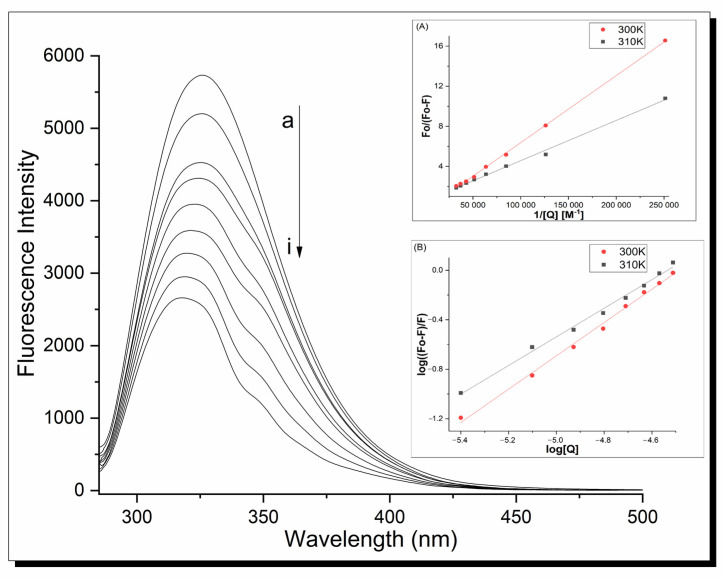
Fluorescence spectra of apo-Tf complex **1** system at 310 K, pH 7.40, λex = 280 nm; inset (**A**) shows modified Stern–Volmer curves; inset (**B**) shows plots of log((F_0_ − F)/F) vs. log[Q] under the same conditions. Analogical spectra were obtained for complex **2**.

Fluorescence spectroscopy was used to determine the mechanism of action and binding constants between proteins and ligands. The fluorescence analysis was performed at λex = 280 nm. In this condition, human serum apo-transferrin shows a strong fluorescence signal at 326 nm, but clear metal complex fluorescence spectra are also registered because of terpyridine’s presence. Therefore, to clarify all interactions, the fluorescence spectra are shown as differential line–{apo-Tf-Cu complex}-{Cu complex} in an appropriate molar ratio. The fluorescence emission spectra of the apo-Tf-Cu systems are shown in Figure 6. Increasing the Cu compound concentration caused a loss of intensity of the fluorescence signal of the protein with a significant blue shift of the maximum emission peak (from 326 nm for apo-Tf to 317 nm for the system in the molar ratio 1:16). This indicates the interaction between the protein and copper complexes and suggests that Trp-214 is located in a more hydrophilic environment.

The modified Stern–Volmer equation (Equation (3)) was made to analyze the protein fluorescence quenching parameters [55].

(3) $$\frac{F_{0}}{F_{0}-F}=\frac{1}{f_{a}K_{SV[Q]}}+\frac{1}{f_{a}}$$

(4) $$K_{q}=\frac{K_{SV}}{\tau_{0}}$$

where F_0_ and F are the fluorescence intensities of the protein in the absence and presence of a quencher, respectively, K_sv_ is the Stern–Volmer quenching constant, K_q_ is the quenching rate constants, [Q] is a quencher concentration and τ0 is the average lifetime of a biomolecule without quencher for apo-Tf τ_0_ =2.5 × 10^−9^ s.

Based on the above relationships, the fluorescence quenching mechanism and K_sv_/K_q_ constants were calculated. All the quenching parameters are listed in Table 5. The data show the dynamic quenching mechanism of the apo-transferrin fluorescence in the interaction with both complexes—the K_sv_ values increase with temperature. Simultaneously, the K_q_ values are higher than the maximum dynamic quenching constants of the other quenchers with the biopolymer (2.0 × 10^10^ M^−1^s^−1^). Therefore, we can consider a combined fluorescence quenching mechanism with the formation of a stable complex between the copper complexes and apo-Tf. The association constants between the protein and ligand as well as several binding sites for the metal complex in the protein were calculated using Equation (5):

(5) $$log\frac{{(F}_{0}-F)}{F}=logK_{a}+nlog[Q]$$

where F_0_ and F are the fluorescence intensities in the absence and presence of the quencher, respectively, K_a_ is the binding constant, n is the number of binding sites, and [Q] is the total concentration of the quencher.

It is observed that the K_a_ values decrease with the temperature increase. This indicates unstable protein–Cu complex adduct formation with a partial decomposition at higher temperatures. The calculated n values approximate to 1 in all the cases indicate one binding site for the ligand in the protein. Under pseudo-physiological conditions (pH 7.40, T = 310 K), the binding constants of the systems are a row of 10^5^–10^6^ M^−1^. This points to a rather strong affinity of the complexes to apo-Tf [56]. In comparison, the association constants of the other metal complexes bound to human serum transferrin are given, e.g., 1.37 × 10^5^ M^−1^ ((η^5^-C_5_H_5_)_2_VCl_2–_hTf), [57] 5.01 × 10^7^ M^−1^ (U(VI)–apo-Tf) [58], or ~1.3 × 10^4^ M^−1^ in the case of the NAMI-A–apo-Tf system [59].

Taking into account the Ross–Subramanian theory based on Equations (6)–(8), the acting forces and thermodynamic parameters of the apo-Tf-Cu systems were determined:

(6) $$ln\frac{{Ka}_{2}}{{Ka}_{1}}=\frac{{∆H}^{0}}{R}\left(\frac{1}{T_{1}}-\frac{1}{T_{2}}\right)$$

(7) $${∆G}^{0}=-RTlnK_{a}$$

(8) $$∆S^{0}=\frac{{∆H}^{0}-{∆G}^{0}}{T}$$

In the equations, ∆H^0^, ∆S^0^, and ∆G^0^ are the enthalpy, free energy, and entropy, respectively, K_a_ is the binding constant at the corresponding temperature, T is the experimental temperature [K], and R—gas constant.

Proteins and other biologically active molecules interact with small ligands via electrostatic forces, hydrophobic and ionic interaction, or van der Waals and hydrogen bonds. Metal complexes can also interact via metal atoms creating coordination bonds. 

According to the Ross–Subramanian theory and both negative ∆H^0^ and ∆S^0^, it is clear that both metal complexes react with apo-transferrin via van der Waals interactions and hydrogen bonds. The negative values of ∆G^0^ are characteristic for spontaneous processes [60]. 

**Figure 7:**
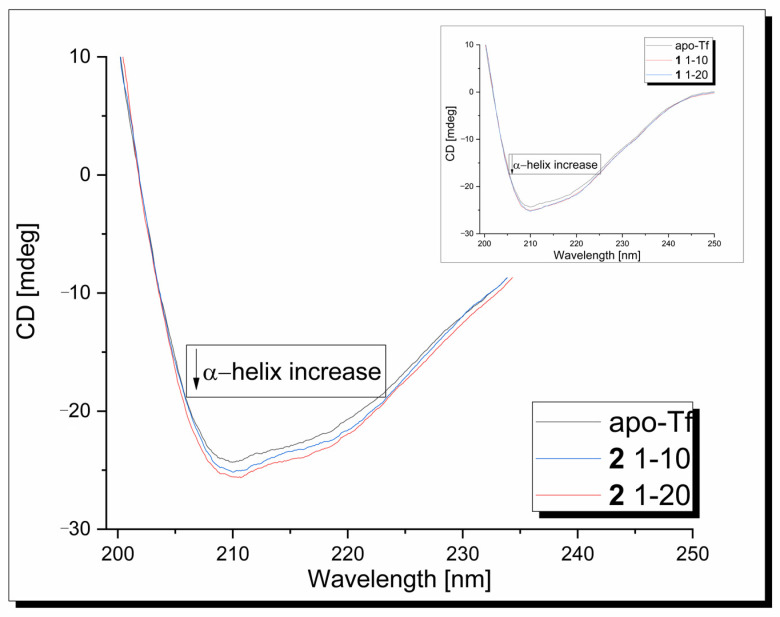
CD spectra of apo-Tf-Cu complexes systems at 310 K, pH 7.40.

Circular dichroism measurements were used to determine the effect of the copper complexes on the protein’s secondary structure. In the CD spectrum of the apo-Tf in the range 190–250 nm, one intensive negative band at 209 nm, assigned to its α-helical structure, is observed (Figure 7). In the second band, broad and weak, its n-π* transition is assigned to around 218 nm [61]. Under experimental conditions, CD signals originating from metal complexes are not generated. The presence of the **1**/**2** compound has a modest effect on the apo-transferrin secondary structure, causing some increase in the band intensity without changes in its position. 

The α-helical content of free apo-Tf at 210 nm was found to be 44.68%. An increase in the α-helicity to 45.84% and 46.36% was observed after the addition of the **1**/**2** copper complex, respectively. This indicates that the binding of the ligands with the protein causes minor conformational changes in the protein, leading to a more stable structure. 

**Figure 8:**
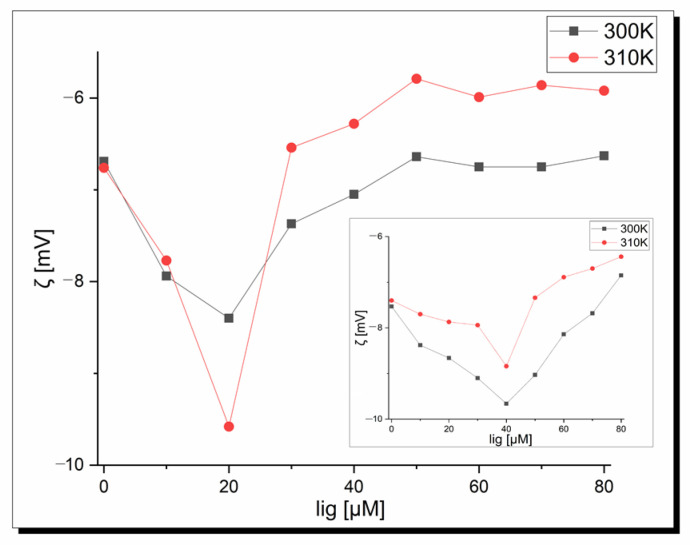
Dependence of the zeta potential of apo-transferrin on the **1** concentration at pH 7.40; inset-**2**.

Zeta potential measurements were performed to confirm the existence of protein–copper complexes interactions and determine the stability of the formed adducts. Figure 8 shows a plot of the zeta potential as a complex concentration function at pH 7.40 depending on temperature (300 K and 310 K). It is seen that pure apo-Tf in pseudo-physiological conditions shows a negative zeta potential independent of the temperature. These negative values indicate hydrophobic forces play a key role in the initial interactions among the apo-Tf and copper complexes. Moreover, a decrease in the zeta potential values was observed in the beginning to tend to the final plateau for higher ligand concentrations. The minimum peak position corresponds to the critical induced aggregation concentration of the copper ligand (Cciac) [62]. This point proves the surface of albumin is full and protein–ligand aggregation occurs [63]. As can be seen in Figure 8, the Cciac point is at 20 µM in both temperatures for complex **1** and at a higher concentration, and 40 µM is observed for complex **2**. The shapes of the curves are similar but the zeta potential value is lower at the Cciac point at 310 K (−8.40 mV vs. −9.58 mV) in the case of complex **1**. All the zeta potential values are higher at 310 K for the apo-Tf-complex **2** system. Based on the theorem that the system is stable when the zeta potential values are higher than ±30 Mv [64], it is seen that under physiological conditions, apo-transferrin-Cu-complex systems show instability, which is very desirable in pharmacology and the design of new drugs and therapies. These results are in agreement with fluorescence studies. In conclusion, temperature affects the zeta potential values but does not influence the Cciac point and stability of the systems.

## 3. Materials and Methods

### 3.1. Chemicals

All standard chemicals and solvents were obtained from commercial suppliers. PTA (1,3,5-triaza-7-phosphaadamantane) and [Cu(MeCN)_4_][PF_6_] were synthesized according to published methods [65,66,67].

Human apo-transferrin was purchased from Sigma-Aldrich Co. (Catalog No. T1147 ≥98% purity, St. Louis, MO, USA). The protein stock solution with a concentration of 10 μM was prepared in PBS buffer (pH = 7.40). Metal complex stock solutions with a concentration of 800 μM were prepared by dissolution in double distilled water. The final protein concentration in samples was equal to 2 μM during the fluorescence experiment and 4 μM and 5 μM in CD and zeta potential techniques, respectively. Protein concentration was calculated using ε_apo-Tf_ = 83,800 M^−1^ cm^−1^ at 280 nm [68].

Analytical Methods. Bruker IFS 1113v (Ettlingen, Germany) or BIO-RAD FTS3000MX (BIO-RAD, Paris, France) instruments (range 4000–400 cm^−1^) were used to measure the IR spectra (abbreviations: vs, very strong; s, strong; m, medium; w, weak; br., broad). The NMR spectra were obtained using a Bruker 500 AMX spectrometer from Bruker BioSpin MRI GmbH, Ettlingen, Germany, in an acetone-*d*_6_ solvent, both at room and low temperatures. The abbreviations used in the spectra are s for singlet, d for doublet, t for triplet, and br. for broad. ^1^H chemical shifts (*δ*) are expressed in ppm relative to Si(Me)_4_, while *δ*(^31^P) shifts are relative to an external H_3_PO_4_ (85% aqueous solution). The Elemental Analyser Vario ELCube (Elementar Analysen Systeme GmbH, Langenselbold, Germany) was used for the determination of C, H, and N contents (Laboratory of Elemental Analysis at Faculty of Chemistry, University of Wrocław, Wrocław, Poland). To determine the solubility of compounds **1** and **2**, we used an automatic pipette to add small amounts of water to a specified amount of each compound. After each addition, we placed the mixture in an ultrasonic bath at 25 °C and checked the transparency of the resulting mixture. We repeated the process until a clear solution was obtained. 

### 3.2. Synthesis of ***1*** and ***2***

Via LAG, compounds were synthesized using [Cu(MeCN)_4_][PF_6_] (0.25 mmol, 93.2 mg), PTA (0.25 mmol, 39.3 mg for **1,** 0.5 mmol, 78.6 mg for **2**), and terpy (0.25 mmol, 58.4 mg) in a 1:1:1 ratio for **1** and 1:2:1 ratio for **2**. The mixture was ground in a mortar for 2 min with 80 µL of MeCN present. Afterward, the microcrystalline yellow solid was washed with cold methanol and dried in the air. Yields for **1** and **2**, based on [Cu(MeCN)_4_][PF_6_], are 75 and 95%, respectively. These compounds are soluble in H_2_O (*S*_25°C_ = 5 and 23 mg∙mL^−1^ for **1** and **2**, respectively), DMSO, MeCN, and acetone, and slightly soluble in methanol, ethanol, and THF. The elemental analysis for **1**: Calculated for C_21_H_23_CuN_6_P_2_F_6_ (MW 598.93): C, 42.11; H, 3.87; N, 14.03. Found: C, 42.35; H, 3.82; N, 14.11. The elemental analysis for **2**: Calculated for C_27_H_35_CuN_9_P_3_F_6_ (MW 756.09): C, 42.89; H, 4.67; N, 16.67. Found: C, 42.71; H, 4.70; N, 16.66. IR for **1** (KBr, cm^−1^): 3435 (br, s), 3064 (m), 2925 (m), 1600 (m), 1574 (m), 1475 (s), 1448 (s), 1416 (m), 1397 (w), 1303 (w), 1289 (m), 1279 (w), 1242 (s), 1185 (vw), 1166 (w), 1102 (w), 1095 (w), 1042 (w), 1016 (vs), 969 (vs), 947 (s), 893 (w), 840 (vs), 772 (vs), 742 (s), 651 (w), 631 (vw), 584 (s), 557 (vs), 466 (w), 451 (w), 433 (vw), 398 (w). IR for **2** (KBr, cm^−1^): 3429 (br, m), 3063 (w), 2954 (m), 2927 (s) 2892 (s), 2880 (s), 2816 (w), 2019 (vw), 1985 (vw), 1760 (vw), 1589 (m), 1574 (m), 1568 (m), 1474 (m), 1465 (w), 1447 (s), 1436 (s), 1416 (s), 1397 (w), 1365 (vw), 1341 (vw), 1329 (vw), 1302 (w), 1288 (s), 1279 (s), 1242 (vs), 1184 (w), 1165 (w), 1102 (s), 1094 (m), 1042 (m), 1016 (vs), 969 (vs), 947 (vs), 910 (w), 893 (m), 875 (s), 842 (vs), 800 (s), 774 (vs), 751 (m), 742 (vs), 730 (m), 650 (w), 631 (w), 618 (vw), 584 (vs), 574 (m), 567 (s), 557 (vs), 477 (vw), 466 (m), 451 (w), 432 (vw), 412 (vw), 398 (m). ^1^H NMR for **1** (500.1 MHz, acetone-*d*_6_, 233K): *δ* 8.99 (br s, 2H, ^6,6″^H, terpy), 8.55 (d, 2H, ^3,3″^H, *J*_4,4″_ = 7.6 Hz, terpy), 8.47 (d, 2H, ^3′,5′^H, *J*_4′_ = 7.6 Hz, terpy), 8.39 (t, 1H, ^4′^H, *J*_3′,5′_ = 7.6 Hz, terpy), 8.20 (dd, 2H, ^4,4″^H, *J*_3,3″_ = *J*_5,5″_ = 7.6 Hz, terpy), 7.75 (dd, 2H, ^5,5″^H, *J*_4,4″_ = 7.6 Hz, *J*_6,6″_ = 5.1 Hz, terpy), 4.38 (br s, 6H, NCH_2_N, PTA), 3.83 (br s, 6H, PCH_2_N, PTA). ^31^P{^1^H} NMR for **1** (202.5 MHz, acetone-*d*_6_, 233K): −93.3 (br s, PTA), −143.7 (septet, PF_6,_ *J*_P-F_ = 710.2 Hz). ^1^H NMR for **2** (500.1 MHz, acetone-*d*_6_, 233K): *δ* 9.04 (d, 2H, ^6,6″^H, *J*_5,5″_ = 4.5 Hz, terpy), 8.59 (d, 2H, ^3,3″^H, *J*_4,4″_ = 7.9 Hz, terpy), 8.52 (d, 2H, ^3′,5^′H, *J*_4′_ = 7.9 Hz, terpy), 8.42 (t, 1H, ^4′^H, *J*_3′,5′_ = 7.9 Hz, terpy), 8.24 (dd, 2H, ^4,4″^H, *J*_3,3″_ = *J*_5,5″_ = 7.9 Hz, terpy), 7.80 (dd, 2H, ^5,5″^H, *J*_4,4″_ = 7.9 Hz, *J*_6,6″_ = 4.5 Hz, terpy), 4.66 and 4.49 (2d, 6H, *J*_AB_ = 12.5 Hz, NC*H*^A^*H*^B^N, PTA’), 4.37 (br s, 6H, PCH_2_N, PTA’), 4.24 (br s, 6H, NCH_2_N, PTA’’), 3.85 (br s, 6H, PCH_2_N, PTA’’). ^31^P{^1^H} NMR for **2** (202.5 MHz, acetone-*d*_6_, 183K): −93.5 (br s, PTA), −143.8 (septet, PF_6,_ *J*_P-F_ = 708.7 Hz). 

Wet synthesis of **1** and **2**. To synthesize compound **1**, a mixture of [Cu(MeCN)_4_][PF_6_] (0.5 mmol, 186.4 mg), PTA (0.5 mmol, 78.6 mg), and terpy (0.5 mmol, 116.7 mg) were dissolved in 50 mL of MeOH in a round-bottom flask. The cloudy solution was stirred at room temperature for 1.5 h, resulting in a yellow solution and microcrystalline, yellow solid. The precipitate was filtered under an inert atmosphere and washed twice with 10 mL of methanol. The yield based on [Cu(MeCN)_4_][PF_6_] was 35%. To synthesize compound **2**, the same process was followed using a stoichiometric ratio of 1:2:1 for [Cu(MeCN)_4_][PF_6_], PTA, and terpy. The yield based on [Cu(MeCN)_4_][PF_6_] was 47%. The obtained solution was filtered and left in a vial to slowly evaporate in the air at room temperature. After 3 days, yellow crystals (including those of X-ray quality) were formed for compound **2**. The elemental analysis for **1**: Calculated for C_21_H_23_CuN_6_P_2_F_6_ (MW 598.93 + 0.5MeOH): C, 41.99; H, 4.10; N, 13.67. Found: C, 42.55; H, 4.23; N, 14.16. The elemental analysis for **2**: Calculated for C_27_H_35_CuN_9_P_3_F_6_ (MW 756.09): C, 42.89; H, 4.67; N, 16.67. Found: C, 42.59; H, 5.04; N, 14.86. IR for **1**. (KBr, cm^−1^): 3435 (br, m), 3062 (w), 2926 (m), 2891 (m), 1710 (vw), 1589 (w), 1574 (m), 1568 (w), 1474 (m), 1447 (m), 1437 (m), 1416 (w), 1397 (w), 1288 (m), 1279 (m),1242 (m), 1184 (w), 1165 (w), 1102 (m), 1094 (w), 1042 (w), 1016 (s), 969 (s), 947 (m), 893 (w), 840 (vs), 774 (vs), 742 (m), 650 (vw), 631 (vw), 584 (s), 557 (s), 466 (vw), 451 (vw), 398 (vw). IR for **2**. (KBr, cm^−1^): 3435 (br, m), 3074 (w), 2947 (m), 1594 (m), 1576 (m), 1564 (w), 1448 (s), 1433 (m), 1422 (w), 1416 (w), 1398 (w), 1369 (vw), 1305 (w), 1294 (m), 1283 (w), 1239 (m), 1191 (vw), 1169 (vw), 1108 (m), 1095 (w), 1072 (vw), 1038 (w), 1019 (s), 999 (w), 987 (w), 971 (s), 951 (m), 946 (m), 895 (w), 843 (vs), 797 (s), 773 (vs), 745 (w), 665 (vw), 652 (w), 628 (vw), 616 (w), 576 (m), 568 (m), 558 (vs), 473 (vw), 458 (vw), 431 (vw), 402 (vw), 397 (vw).

### 3.3. X-ray Crystallography

C_27_H_35_CuF_6_N_9_P_3_, *M* = 756.09, crystal system: monoclinic, space group: *P*2_1_/*n*, *a* = 10.4364(3) Å, *b* = 13.7629(4) Å, *c* = 21.5413(5) Å, β = 98.343(3)°, V = 3061.34(15) Å^3^, *Z* = 4, T = 100(2) K, crystal size: 0.33 × 0.29 × 0.25 mm, *ρ*_c_ = 1.640 g·cm^−3^, μ = 3.150 mm^−1^, absorption correction: analytical, T_min_ = 0.706, T_max_ = 0.860, θ_max_ = 75.785°, reflections: 30,003, independent: 6287, *R*_int_ = 0.0195, *R*_1_ = 0.0331, *wR*_2_ = 0.0877, GoF = 1.057.

Crystallographic data (excluding structure factors) for the structure reported in this paper have been deposited with the Cambridge Crystallographic Data Centre as supplementary publication no. CCDC-2291627 (**2**). Copy of the data can be obtained free of charge on application to the CCDC, 12 Union Road, Cambridge CB2 1EZ, UK [+44-1223/336033; e-mail: deposit@ccdc.cam.ac.uk].

### 3.4. X-ray Crystal Structure Determination

Single-crystal X-ray diffraction data were collected at XtaLAB Synergy R, DW system, four-circle diffractometer with Cu Kα radiation, and Hybrid Pixel Array Detector (Rigaku, Tokyo, Japan). Measurement was carried out at 100(2) K. Programs used for data collection and data reduction CrysAlisPro 1.171.42.50a (Rigaku Oxford Diffraction, Yarnton, Oxfordshire, UK. 2022) [69]. Structures were solved by direct methods and then refined by a full-matrix least squares method with the SHELX-2018/3 [70] program with anisotropic thermal parameters for nonhydrogen atoms. Molecular graphics were prepared with the XP [71] and Diamond [72] programs. Data for publication were prepared with the programs SHELX-2018/3 [70] and PLATON [73]. 

### 3.5. Cell Cultures

Cytotoxic studies carried out on cell cultures of normal human dermal fibroblasts (NHDFs; PromoCell, C-12302^®^, Heidelberg, Germany), human lung carcinoma (A549; ATCC, Manassas, VA, USA, No CCL-185^®^), human breast adenocarcinoma (MCF-7, ATCC, HTB-22) and human cervix carcinoma (HeLa; ATCC, No CCL-2^®^). All cell cultures were cultured in Dulbecco’s Modified Eagle Medium (DMEM) (Thermo Fisher Scientific, Waltham, MA, USA) containing 4.5 g/L d-glucose, 1% 4 nM l-glutamine, 2% 100 U/mL streptomycin, 100 μg/mL penicillin, and 10% fetal bovine serum (FBS) obtained from Sigma-Aldrich.

### 3.6. Cytotoxicity Assay

The conducted test consisted of enzymatic reduction of soluble tetrazolium salt in metabolically active cells into insoluble purple formazan (MTT test). The cultivation was carried out in a SafeGrow Pro (EuroClone, Pero, Italy) incubator at 37 °C with a constant flow of 5% CO_2_ and constant humidity. After cell lines were obtained from the culture flasks by enzymatic digestion with trypsin (0.25%) and EDTA (0.02%) (Sigma, St. Louis, MO, USA), they were applied to Eppendorf 96-well culture plates. A total of 100 μL of the cell suspension at a density of 3 × 10^3^ was added to each well. The cells were left for 24 h and then the culture medium was replaced with a new one. The test substances in following concentrations—0.1, 1, 2, 5, 10, 20, 50, 100, 200 μM/mL were added to the prepared samples and incubated for 72 h. For the negative control sample, we used untreated cells. The positive control was a mitomycin C solution with a concentration of 2 μg/mL. As for the blank sample, we used the culture medium with the same composition mentioned above. A solution of MTT (3-(4,5-dimethylthiazol-2-yl)-2,5-diphenyltetrazolium bromide) with a concentration of 1 mg/mL was prepared (according to Annex C of PN-EN ISO 10993-5:2009) [74]. The solvent was a MEM culture medium without any dyes and phenol red. The samples were incubated for 2 h, after which the MTT solution was removed and 100 μL of isopropyl alcohol (Stanlab, Lublin, Poland) was added to each well. The contents of the wells were mixed, the plate was placed in a Multiskan GO spectrometer (ThermoFisher, Waltham, MA, USA), and the absorbance value was read at a wavelength of 570 nm. The viability of the cells was calculated according to the following formula:

Viability [%] = 100% × average optical density of treated cells/average optical density of untreated cells.

The results are based on four trials that were conducted independently.

### 3.7. Apo-Transferrin Interactions

#### 3.7.1. Fluorescence Measurements

The fluorescence emission spectra were performed on the Jasco 8200 spectrofluorimeter (Tokyo, Japan) in the range 285–500 nm and at λex = 280 nm. All measurements were conducted in PBS buffer solution at 300 K and 310 K using a 1.0 cm quartz cell. The slit of widths of ex and em was set at 5 nm. Due to the strong fluorescence signal of copper complexes in the measuring range, all fluorescence spectra are shown and calculated as differences spectra. The fluorescence experiment was performed by titration of an apo-transferrin sample with an appropriate volume of the copper complex. Fluorescence spectra were recorded after 5 min. incubation. The final concentration of the protein and ligand during the titration experiment was calculated using the following relations:

$$[\mathrm{protein}]=\frac{{\left[protein\right]}_{0}V_{0}}{V_{0}+{\sum V}_{L}},$$

$$[\mathrm{ligand}]=\frac{{\left[lig\right]}_{0}{\sum V}_{L}}{V_{0}+{\sum V}_{L}},$$

where [protein]_0_ and V_0_ are the initial concentration and volume of the protein solution, respectively, the [lig]_0_ is stock ligand solution concentration, and $\sum V_{L}$ is the total volume of the ligand added during the titration experiment. 

#### 3.7.2. Circular Dichroism Spectroscopy

A circular dichroism experiment was performed using the Jasco J-1500 CD spectropolarimeter. Spectra were registered in the range 190–250 nm, by 0.98 mm quartz cell, after 24 h reagent incubation at 310 K. The final concentration of the metal complex was 40 and 80 µM.

#### 3.7.3. Zeta Potential Measurements

The zeta potential measurements were performed on Zetasizer–Nano-ZS (Malvern Instruments, Malvern, UK) at 310 K, after 5 min incubation, using a polycarbonate U-shaped cell with gold-plated electrodes. The final values are shown by taking the average of five measurements.

## 4. Conclusions

In summary, we present a rapid, straightforward, and energy-efficient self-assembly method for synthesizing water-soluble, heteroleptic copper(I) complexes, specifically, [Cu(terpy)(PTA)][PF_6_] (**1**) and [Cu(terpy)(PTA)_2_][PF_6_] (**2**). These products are characterized as stable yellow microcrystalline solids, analyzed through FT-IR, ^1^H, and ^31^P{^1^H} NMR spectroscopy, elemental analysis, and X-ray diffraction (for **2**). The X-ray analysis revealed a bidentate coordination mode of terpyridine and a distorted tetrahedral coordination sphere around the Cu(I) center. The variable-temperature NMR tests indicated dynamic properties for both the terpyridine and PTA ligands in compounds **1** and **2**. 

Compounds **1** and **2**, though unsuitable for LEC device production due to weak emission at room and low temperatures, exhibit promising biological properties. Specifically, compound **1** demonstrates greater effectiveness against cancer cells than compound **2**, with no significant toxicity to normal skin cells. Compared to the other tested compounds, complexes **1** and **2** show significantly lower IC50 values against various cancer cells, suggesting their potential as anticancer agents. While cisplatin also exhibits cytotoxicity, it lacks selectivity, posing a risk to normal cells. Furthermore, both compounds surpass the analog compound [Ag(terpy)(PTA)][NO_3_] in terms of anticancer activity and lower toxicity to normal cells. Briefly, the reported cytotoxic activity of **1** and **2** surpasses that of cisplatin by up to 30 times while demonstrating toxicity towards normal cells that is six times lower. These findings highlight the potential of the tested compound as a promising chemotherapeutic agent in anticancer therapy. In addition, the interactions between the representative complexes and apo-transferrin were investigated by circular dichroism (CD), fluorescence, and UV-Vis spectroscopy. Fluorescence and zeta potential studies showed a strong interaction between apo-transferrin—**1**/**2** copper complexes and the instability of formed systems under physiological conditions. Moreover, the CD analysis proved slight changes in protein secondary structure. All these observations suggest that the apo-transferrin may be good for the copper complexes’ carrier and functions as the Cu compounds’ reservoir for therapeutic purposes.

## Figures and Tables

**Table 1:** Selected geometrical parameters for **2**.

Bond Distances [Å]		Valence Angles [°]	
Cu1-P11	2.1854(5)	P11-Cu1-P21	119.48(2)
Cu1-P21	2.2126(5)	P11-Cu1-N31	118.43(5)
Cu1-N31	2.0785(16)	P11-Cu1-N41	117.71(5)
Cu1-N41	2.0674(15)	P21-Cu1-N31	104.86(5)
		P21-Cu1-N41	109.43(5)
		N31-Cu1-N41	79.87(6)

**Table 2:** Crystallographic data for **2**.

Formula	C_27_H_35_CuF_6_N_9_P_3_
Moiety formula	[Cu(C_15_H_11_N_3_){P(CH_2_)_6_N_3_}_2_][PF_6_]
FW [g·mol^−1^]	756.09
*T* [K]	100(2)
Wavelength [Å]	1.54184
Cryst. Syst.	Monoclinic
Space group	*P*2_1_/*n*
*a* [Å]	10.4364(3)
*b* [Å]	13.7629(4)
*c* [Å]	21.5413(5)
β [°]	98.343(3)
V [Å^3^]	3061.34(15)
*Z*	4
Calc *d* [g·cm^−3^]	1.640
µ [mm^−1^]	3.150
T_min_/T_max_	0.706/0.860
θ range [°]	3.823–75.785
Reflns collected	30003
Indep refunds (Rint)	6287 (0.0195)
GOF on *F^2^*	1.057
Final R1/wR2 indices	0.0331/0.0877
CCDC No.	2291627

**Table 3:** Indices for geometrical shapes of four-coordinate systems.

	THC_DA_/100 [43]	FCGP/100 [44]	τ_4_ [45]
Tetrahedron	1.00	0.00	1.00
Trigonal pyramid	0.00	1.00	0.85
[Cu(terpy)(PTA)_2_]^+^	0.32	0.45	0.87

**Table 4:** Cytotoxicity of **1**, **2**, and reference compounds expressed in IC_50_ values (μM).

Cell Line	1	2	[Cu(MeCN)_4_][PF_6_]	terpy	PTA	[Ag(terpy)(PTA)][NO_3_] [30]	Cisplatin [30]
NHDF	152.36 ± 2.82	171.72 ± 4.77	24.75 ± 3.64	1.71 ± 0.95	127.39 ± 7.84	50.07 ± 4.74	16.65 ± 2.17
A549	0.64 ± 0.35	49.87 ± 1.14	0.89 ± 1.14	0.75 ± 0.23	141.84 ± 3.62	1.67 ± 2.53	33.30 ± 4.23
HeLa	0.31 ± 0.61	5.09 ± 0.79	5.02 ± 1.72	0.33 ± 0.41	110.73 ± 4.77	1.67 ± 3.21	16.65 ± 3.19
MCF-7	1.29 ± 2.47	0.23 ± 1.39	22.02 ± 2.49	0.38 ± 0.17	148.19 ± 7.43	26.75 ± 2.81	33.30 ± 4.28

**Table 5:** Binding and thermodynamic parameters of the interaction of apo-Tf-Cu complex systems.

Compound	T [K]	Ksv [M^−1^]	Kq [M^−1^s^−1^]	Ka [M^−1^]	n	∆H [kJ/mol]	∆S [J/mol]	∆G [kJ/mol]
**1**	300	6.26 × 10^3^	2.50 × 10^12^	5.39 × 10^5^	1.25	−69.30	−121.28	−32.92
	310	2.26 × 10^4^	9.02 × 10^12^	2.20 × 10^5^	1.16			−31.70
**2**	300	4.86 × 10^3^	1.94 × 10^12^	1.08 × 10^6^	1.34	−141.20	−355.17	−34.65
	310	1.40 × 10^4^	5.60 × 10^12^	1.74 × 10^5^	1.16			−31.10

## Data Availability

Data are contained within the article and Supplementary Materials.

## Supplementary Materials

The following supporting information can be downloaded at: https://www.mdpi.com/article/10.3390/molecules29050945/s1.
