# Supplementary material for: Design, Synthesis, and Anti-Cancer Evaluation of Novel Water-Soluble Copper(I) Complexes Bearing Terpyridine and PTA Ligands
Source: Molecules. 2024 Feb 21;29(5):945. doi: 10.3390/molecules29050945 (PMC10934221; doi:10.3390/molecules29050945)

checkCIF/PLATON report

Structure factors have been supplied for datablock(s) k8_abs

THIS REPORT IS FOR GUIDANCE ONLY. IF USED AS PART OF A REVIEW PROCEDURE FOR PUBLICATION, IT SHOULD NOT REPLACE THE EXPERTISE OF AN EXPERIENCED CRYSTALLOGRAPHIC REFEREE.

No syntax errors found. CIF dictionary Interpreting this report

Datablock: k8_abs

Bond precision:	C-C = 0.0030 A		Wavelength=1.54184
Cell:	a=10.4364(3)	b=13.7629(4)	c=21.5413(5)
	alpha=90	beta=98.343(3)	gamma=90
Temperature:	100 K		

	Calculated	Reported
Volume	3061.34(15)	3061.34(15)
Space group	P 21/n	P 21/n
Hall group	-P 2yn	-P 2yn
Moiety formula	C27 H35 Cu N9 P2, F6 P	C27 H35 Cu N9 P2, F6 P
Sum formula	C27 H35 Cu F6 N9 P3	C27 H35 Cu F6 N9 P3
Mr	756.10	756.09
Dx,g cm-3	1.640	1.640
Z	4	4
Mu (mm-1)	3.150	3.150
F000	1552.0	1552.0
F000'	1552.17	
h,k,lmax	13,17,27	13,17,27
Nref	6366	6287
Tmin,Tmax	0.630,0.807	0.706,0.860
Tmin'	0.547	

Correction method= # Reported T Limits: Tmin=0.706 Tmax=0.860
AbsCorr = ANALYTICAL

Data completeness= 0.988 Theta(max)= 75.785

R(reflections)= 0.0331(5776) wR2(reflections)= 0.0878(6287)

S = 1.058 Npar= 415

The following ALERTS were generated. Each ALERT has the format
test-name_ALERT_alert-type_alert-level.
Click on the hyperlinks for more details of the test.

Alert level C

```
PLAT230_ALERT_2_C Hirshfeld Test Diff for    P21      --C21      .        6.3 s.u.
```

Alert level G

```
PLAT244_ALERT_4_G Low    'Solvent' Ueq as Compared to Neighbors of         P3 Check
PLAT883_ALERT_1_G No Info/Value for _atom_sites_solution_primary .     Please Do !
PLAT912_ALERT_4_G Missing # of FCF Reflections Above STh/L=  0.600         80 Note
PLAT941_ALERT_3_G Average HKL Measurement Multiplicity ..........        4.8 Low
PLAT978_ALERT_2_G Number C-C Bonds with Positive Residual Density.          6 Info
```

0 **ALERT level A** = Most likely a serious problem - resolve or explain
0 **ALERT level B** = A potentially serious problem, consider carefully
1 **ALERT level C** = Check. Ensure it is not caused by an omission or oversight
5 **ALERT level G** = General information/check it is not something unexpected

1 ALERT type 1 CIF construction/syntax error, inconsistent or missing data
2 ALERT type 2 Indicator that the structure model may be wrong or deficient
1 ALERT type 3 Indicator that the structure quality may be low
2 ALERT type 4 Improvement, methodology, query or suggestion
0 ALERT type 5 Informative message, check

It is advisable to attempt to resolve as many as possible of the alerts in all categories. Often the minor alerts point to easily fixed oversights, errors and omissions in your CIF or refinement strategy, so attention to these fine details can be worthwhile. In order to resolve some of the more serious problems it may be necessary to carry out additional measurements or structure refinements. However, the purpose of your study may justify the reported deviations and the more serious of these should normally be commented upon in the discussion or experimental section of a paper or in the "special_details" fields of the CIF. checkCIF was carefully designed to identify outliers and unusual parameters, but every test has its limitations and alerts that are not important in a particular case may appear. Conversely, the absence of alerts does not guarantee there are no aspects of the results needing attention. It is up to the individual to critically assess their own results and, if necessary, seek expert advice.

Publication of your CIF in IUCr journals

A basic structural check has been run on your CIF. These basic checks will be run on all CIFs submitted for publication in IUCr journals (*Acta Crystallographica*, *Journal of Applied Crystallography*, *Journal of Synchrotron Radiation*); however, if you intend to submit to *Acta Crystallographica Section C* or *E* or *IUCrData*, you should make sure that full publication checks are run on the final version of your CIF prior to submission.

Publication of your CIF in other journals

Please refer to the *Notes for Authors* of the relevant journal for any special instructions relating to CIF submission.

PLATON version of 28/11/2022; check.def file version of 28/11/2022

Datablock k8_abs - ellipsoid plot

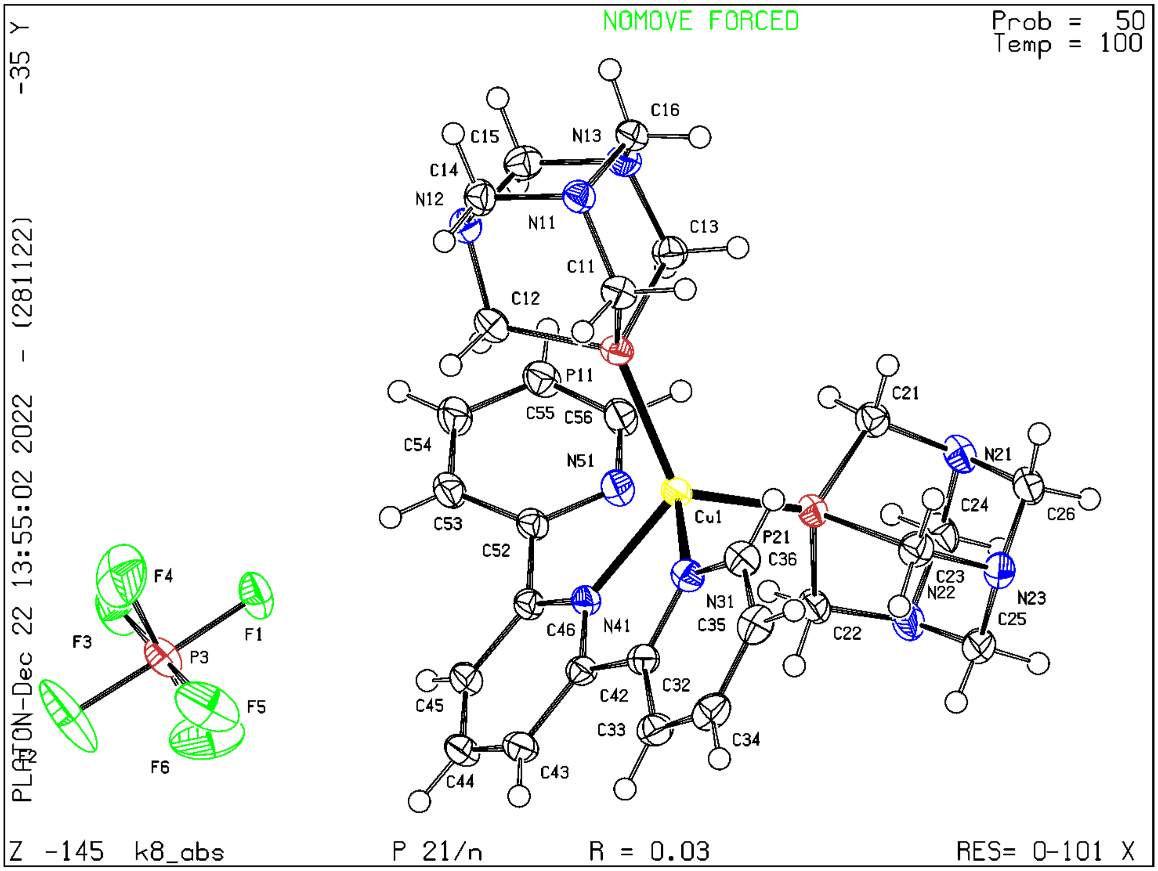

Supplement: Supplementary file 1 [file molecules-29-00945-s001.zip › k8_abs_checkcif.pdf]
