# Supplementary material for: Design, Synthesis, and Anti-Cancer Evaluation of Novel Water-Soluble Copper(I) Complexes Bearing Terpyridine and PTA Ligands
Source: Molecules. 2024 Feb 21;29(5):945. doi: 10.3390/molecules29050945 (PMC10934221; doi:10.3390/molecules29050945)
Supplement: Supplementary file 1 [file molecules-29-00945-s001.zip › Supplementary Data.pdf]

Supplementary Material

Design, Synthesis, and Anti-Cancer Evaluation of Novel Water-Soluble Copper(I) Complexes Bearing Terpyridine and PTA Ligands

Piotr Smoleński [1,*], Urszula Śliwińska-Hill [2], Anna Kwiecień [2], Joanna Wolińska [3] and Dominik Poradowski [3]

[1] Faculty of Chemistry, University of Wrocław, F. Joliot-Curie 14, 50-383 Wrocław, Poland
[2] Department of Basic Chemical Sciences, Faculty of Pharmacy, Wrocław Medical University, Borowska 211a, 50-556 Wrocław, Poland; urszula.sliwinska-hill@umw.edu.pl (U.Ś.-H.)
[3] Department of Biostructure and Animal Physiology, Faculty of Veterinary Medicine, Wrocław University of Environmental and Life Sciences, Kożuchowska 1, 51-631 Wrocław, Poland; 118689@student.upwr.edu.pl (J.W.)
* Correspondence: piotr.smolenski@uwr.edu.pl

Table of Contents

Figure S1. PXRD patterns of compound **2** 3
Figure S2. PXRD patterns of compound **1** 3
Figure S3. IR spectrum of **1** (LAG method) 4
Figure S4. IR spectrum of **1** (wet method) 4
Figure S5. IR spectrum of **2** (LAG method) 5
Figure S6. IR spectrum of **2** (wet method) 5
Figure S7. ^{1}H NMR spectrum of **1** (0 - 12 ppm range with integrals, 233 K) 6
Figure S8. ^{1}H NMR spectrum of **1**(298 K) 7
Figure S9. ^{1}H NMR spectrum of **1**(273 K) 7
Figure S10. ^{1}H NMR spectrum of **1**(253 K) 8
Figure S11. ^{1}H NMR spectrum of **1**(233 K) 7
Figure S12. ^{31}P{^{1}H} NMR spectrum of **1** (233 K) 9
Figure S13. ^{1}H NMR spectrum of **2** (0 - 12 ppm range with integrals, 233 K) 10
Figure S14. ^{1}H NMR spectrum of **2** (298 K) 11
Figure S15. ^{1}H NMR spectrum of **2** (253 K) 11
Figure S16. ^{1}H NMR spectrum of **2** (233 K) 12
Figure S17. ^{1}H NMR spectrum of **2** (213 K) 12
Figure S18. ^{1}H NMR spectrum of **2** (193 K) 13
Figure S19. ^{1}H NMR spectrum of **2** (183 K) 13
Figure S20. ^{31}P{^{1}H} NMR spectrum of **2 (**233 K). 14
Table S1. Proposed stacking interaction between aromatic rings 15
Table S2. Analysis of C-H···π interactions. 15
Table S3. Analysis of potential C-H···X hydrogen bonds. 16

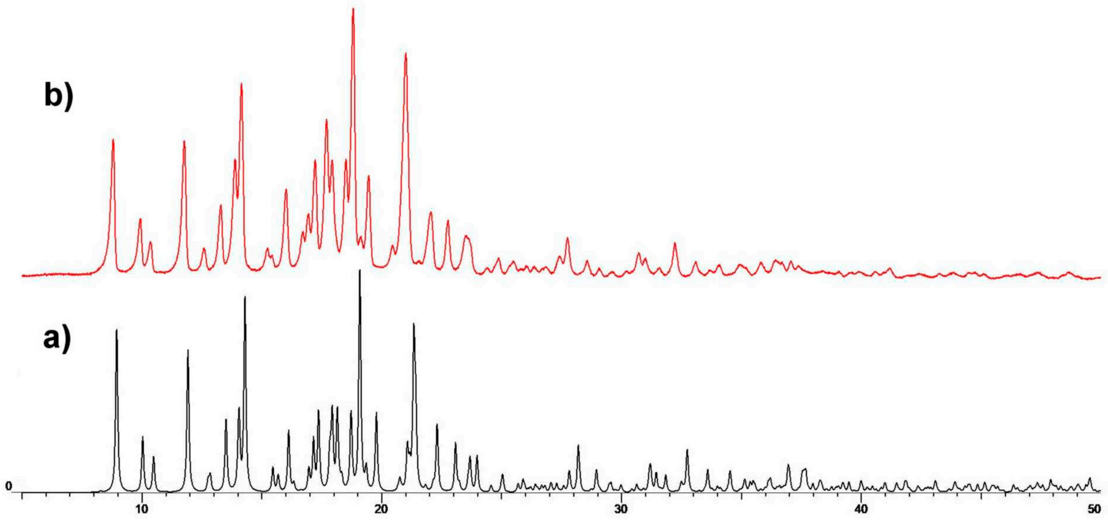

Figure S1. PXRD patterns of compound **2**: (a) calculated from the single crystal structure (black line), (b) bulk microcrystalline product (red line).

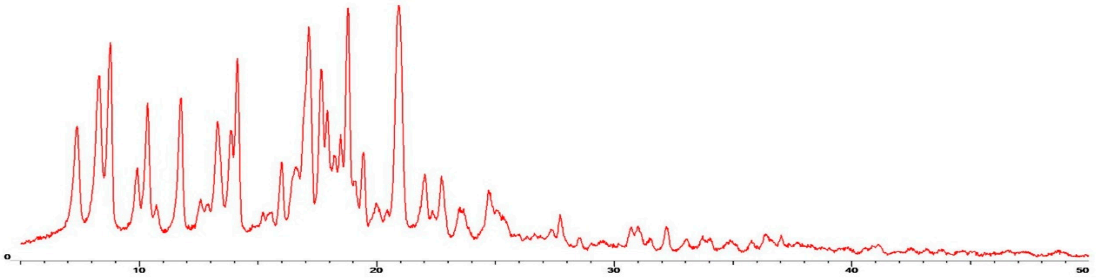

Figure S2. PXRD patterns of compound **1** (bulk microcrystalline product).

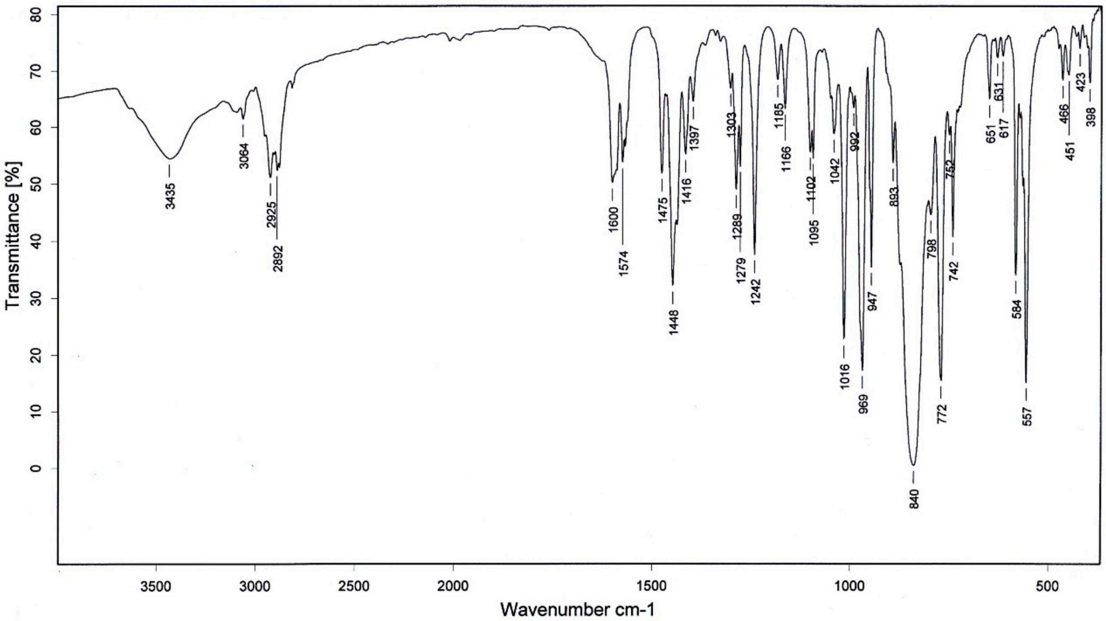

Figure S3. IR spectrum of **1** (LAG method).

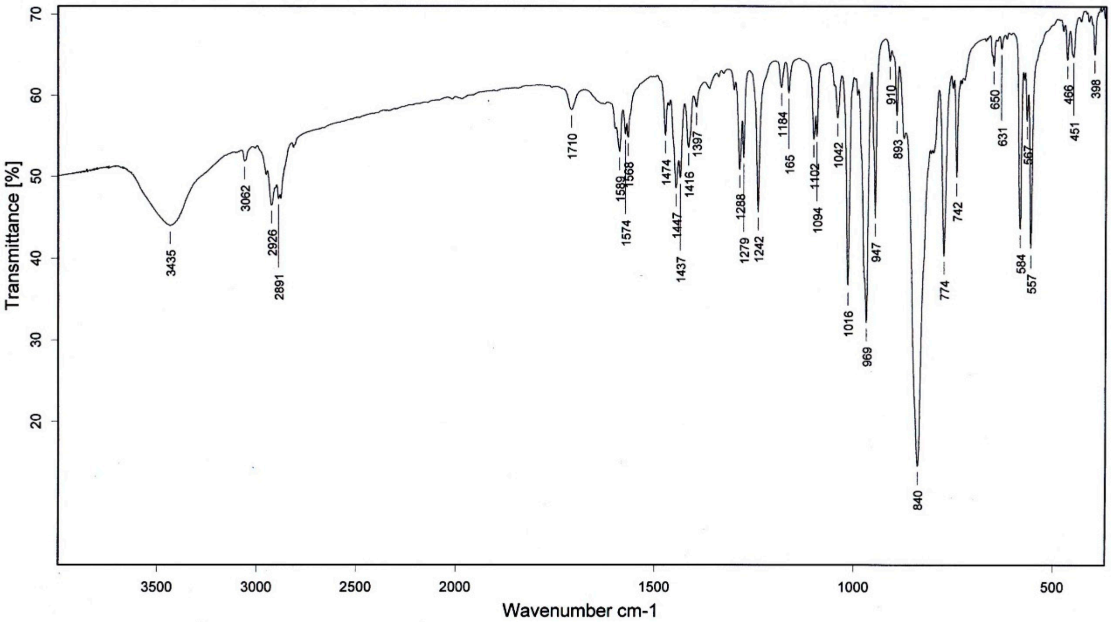

Figure S4. IR spectrum of **1** (wet method).

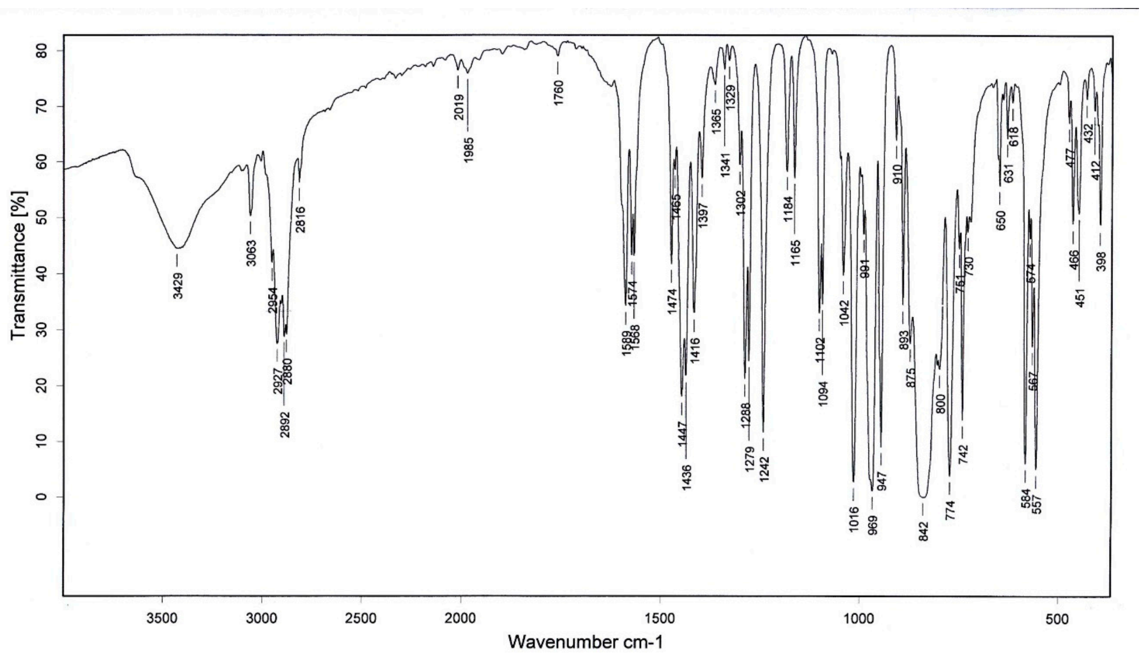

Figure S5. IR spectrum of **2** (LAG method).

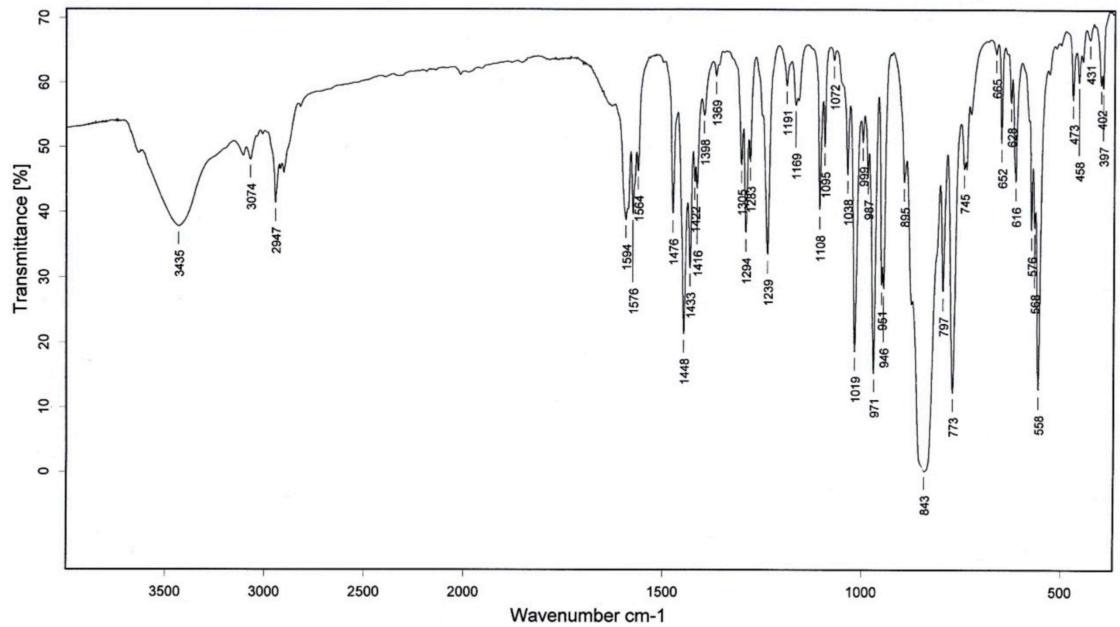

Figure S6. IR spectrum of **2** (wet method).

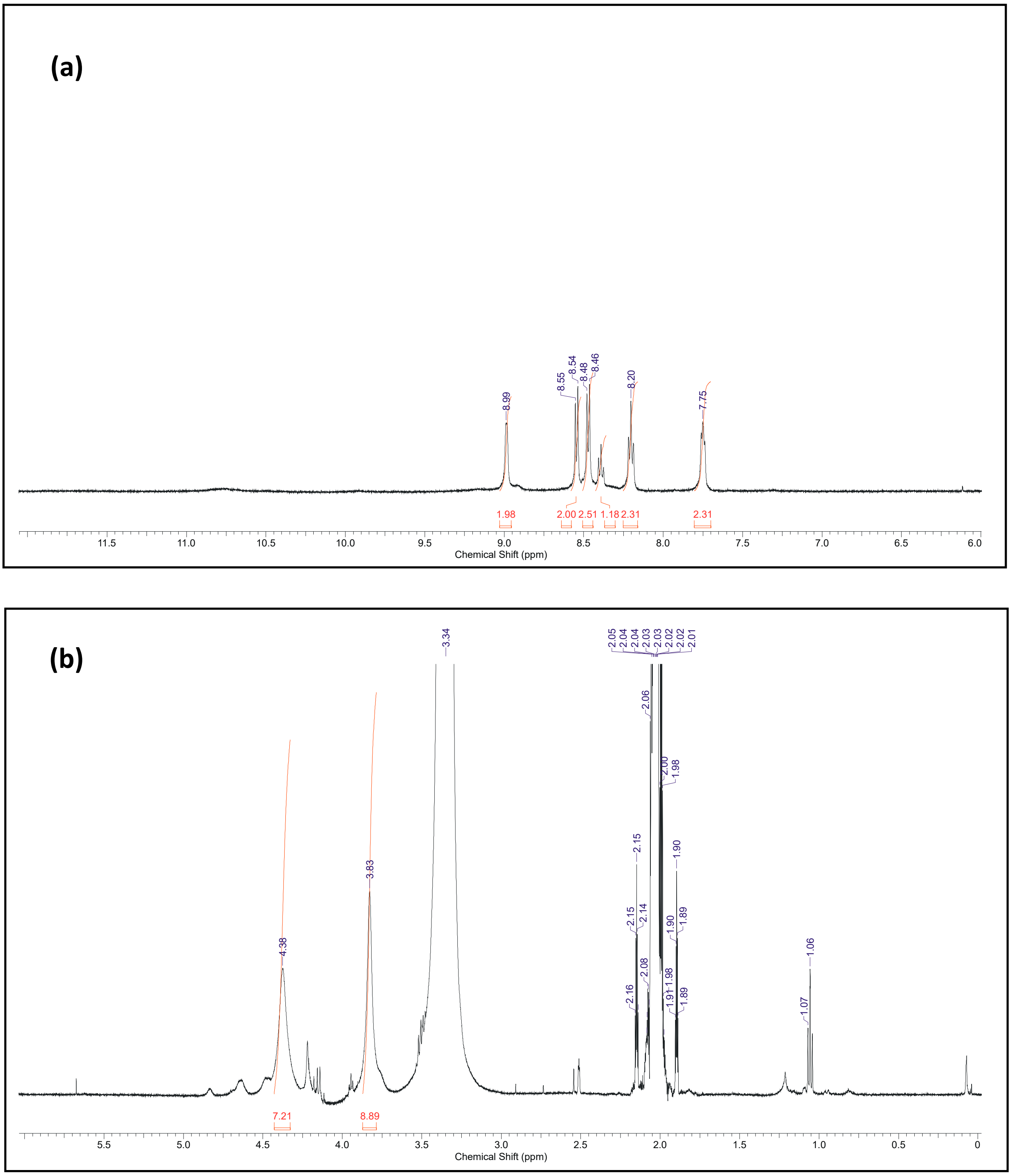

Figure S7. ^{1}H NMR spectrum of **1** [12 - 6 (a) and 6 - 0 ppm (b) range, with integrals, 233 K, acetone-d_6].

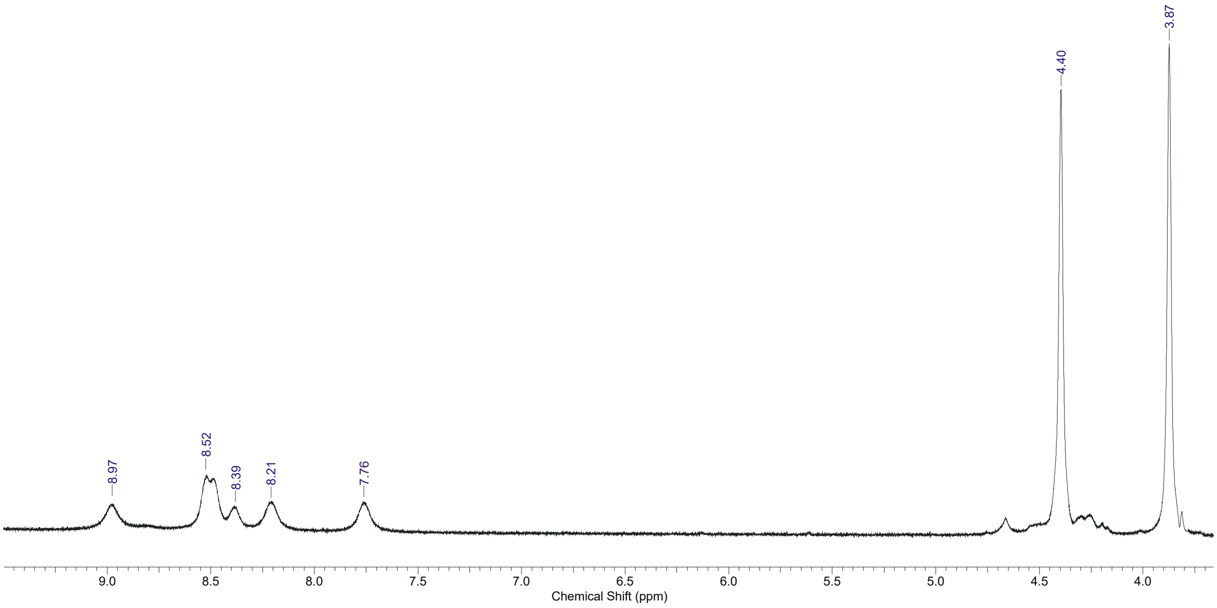

Figure S8. ^{1}H NMR spectrum of **1**(298 K).

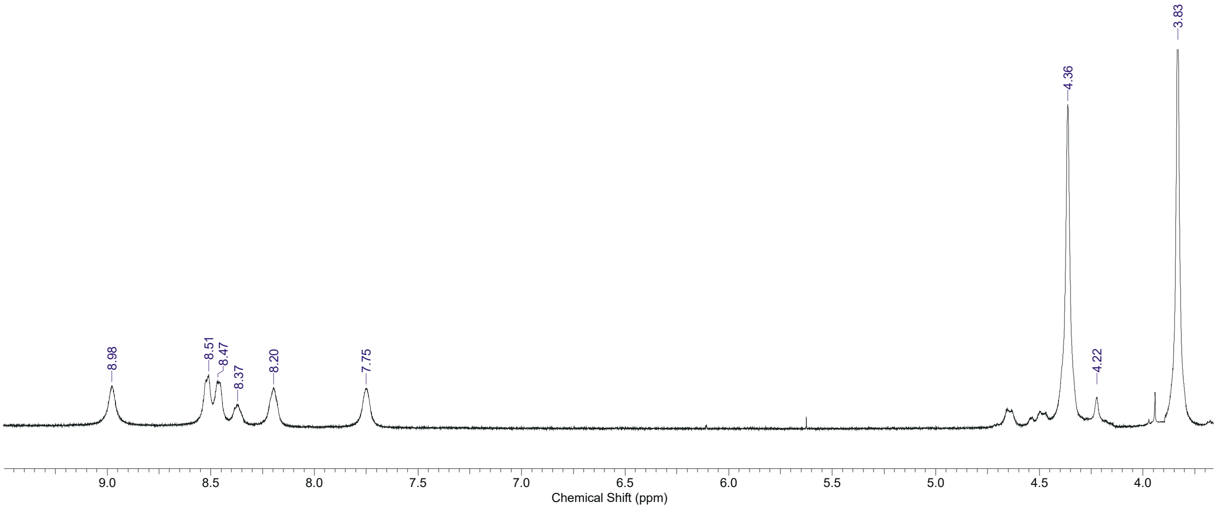

Figure S9. ^{1}H NMR spectrum of **1**(273 K).

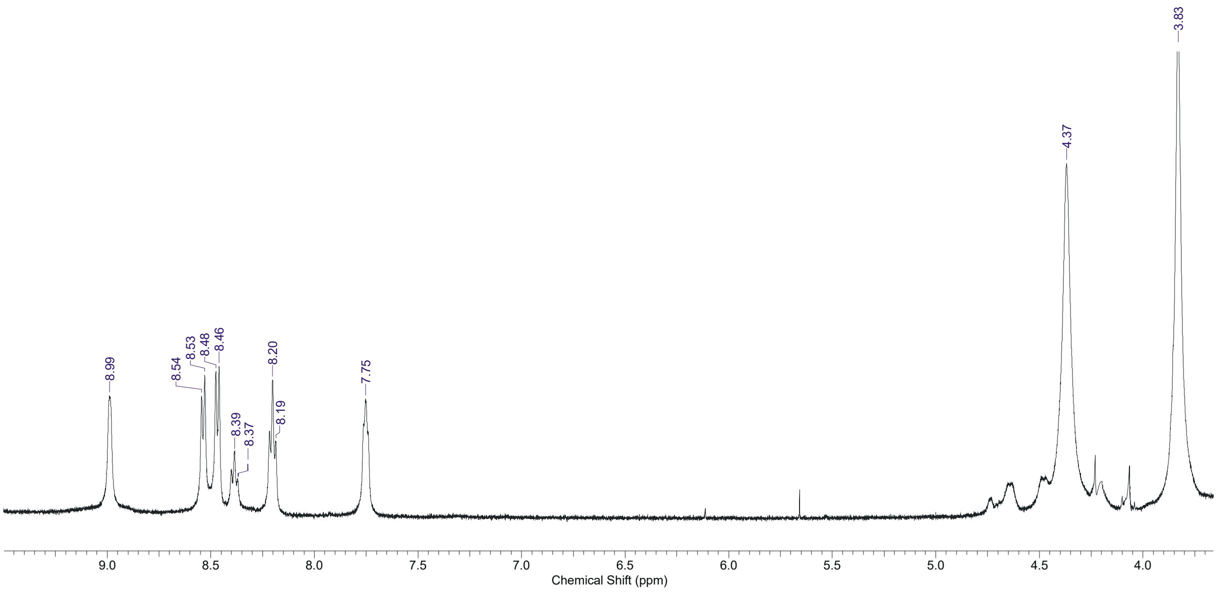

Figure S10. ^{1}H NMR spectrum of **1**(253 K).

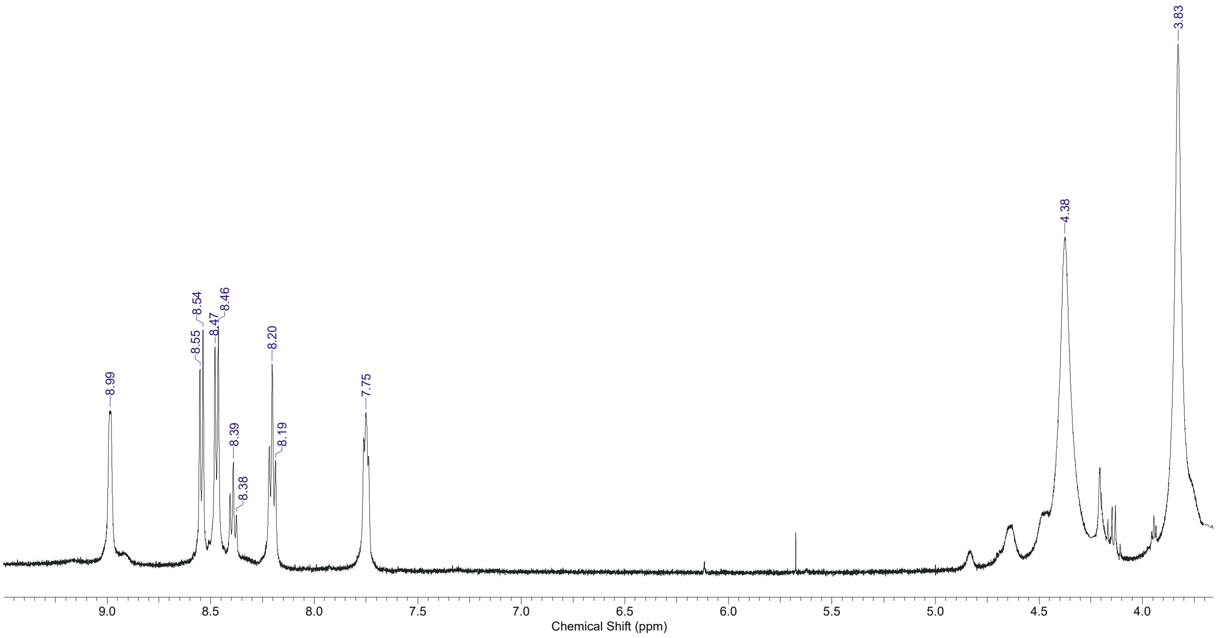

Figure S11. ^{1}H NMR spectrum of **1**(233 K).

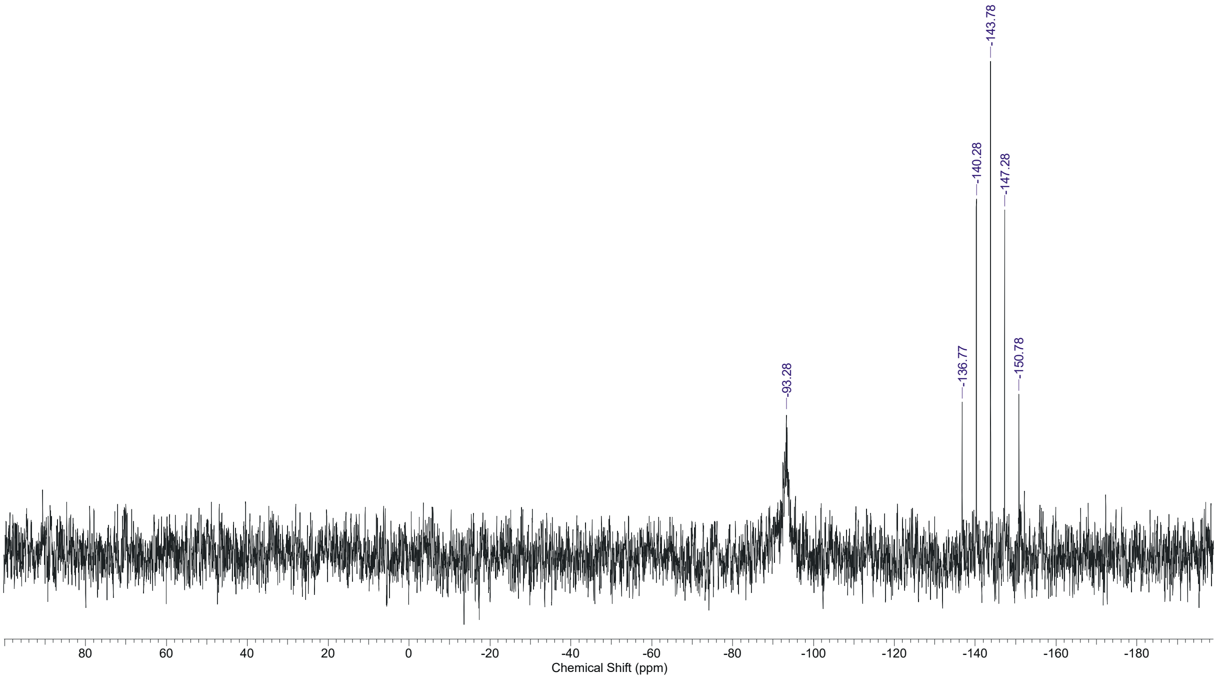

Figure S12. $^{31}P\{^1H\}$ NMR spectrum of **1** (233 K).

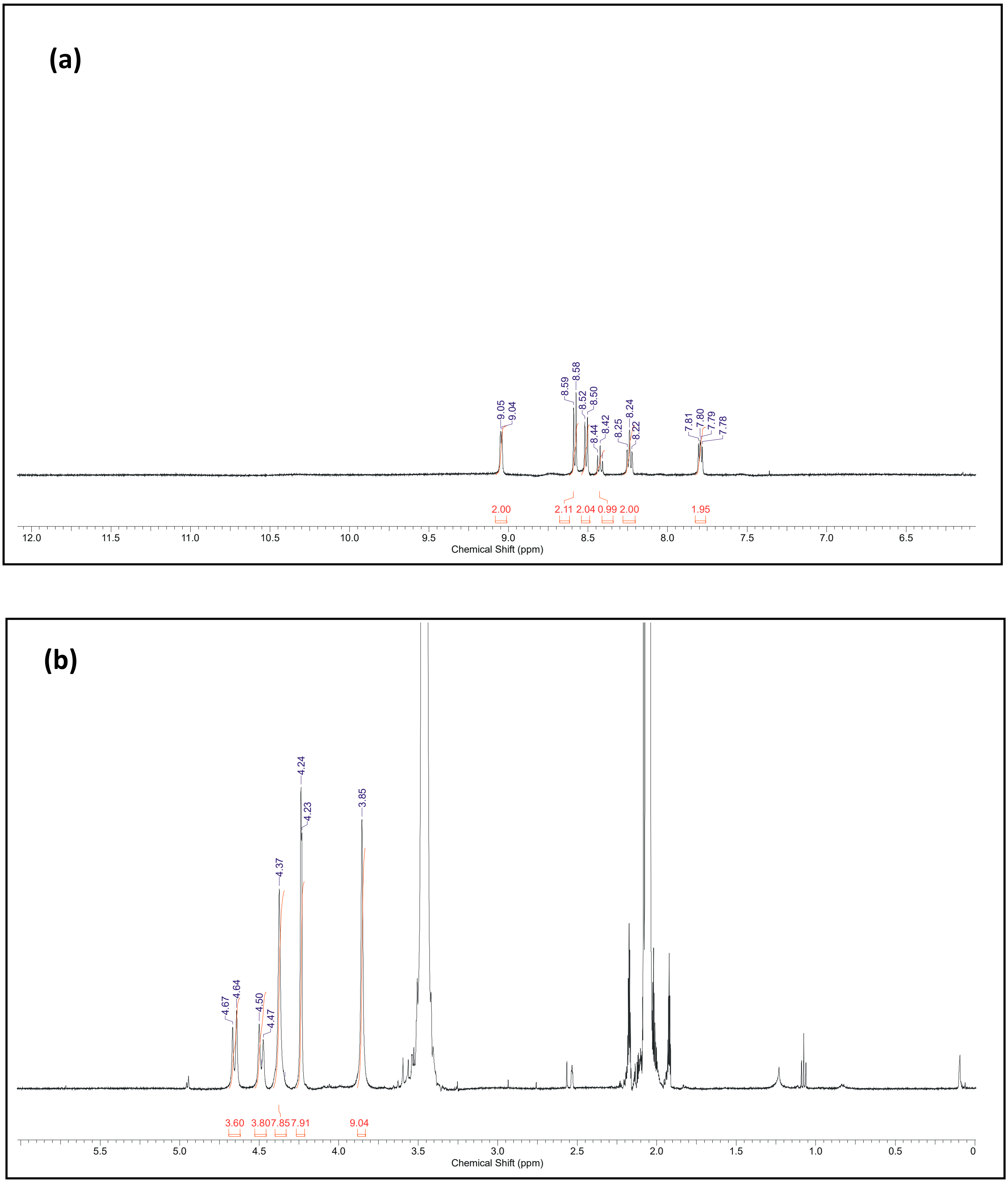

Figure S13. ^{1}H NMR spectrum of **2** [12 - 6 (a) and 6 - 0 (b) ppm range, with integrals, 233 K, acetone-*d*$_6$].

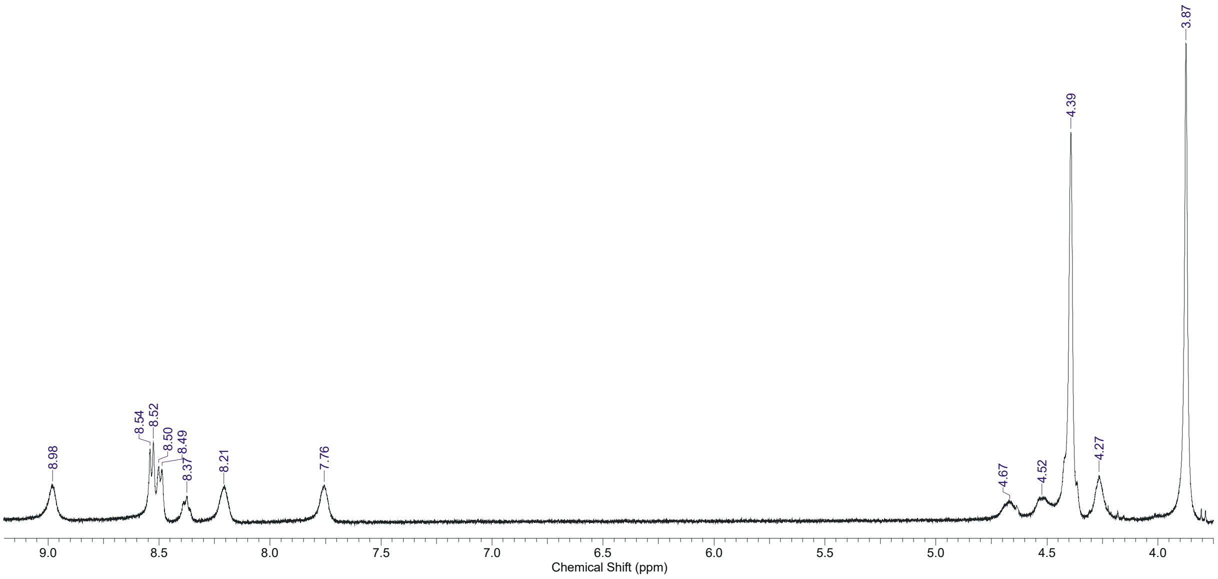

Figure S14. ^{1}H NMR spectrum of **2** (298 K)

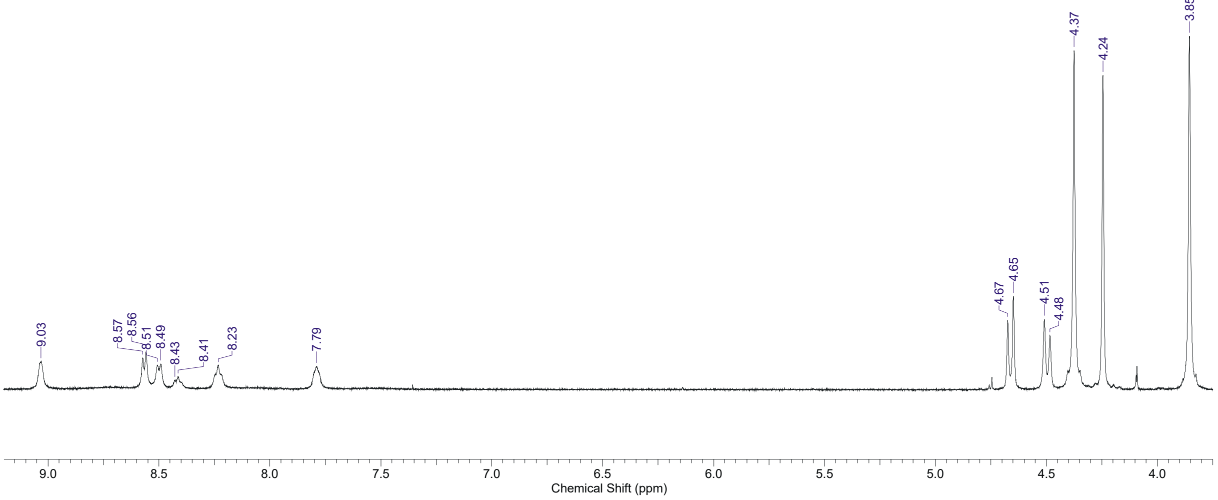

Figure S15. ^{1}H NMR spectrum of **2** (253 K).

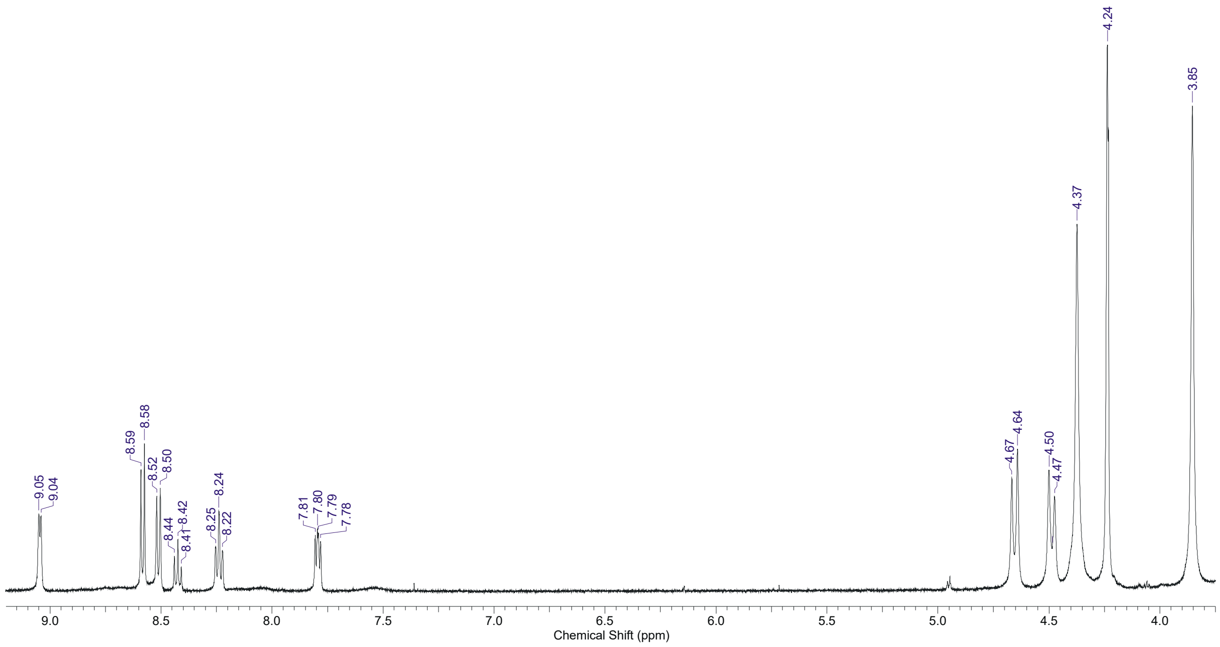

Figure S16. ^{1}H NMR spectrum of **2** (233 K).

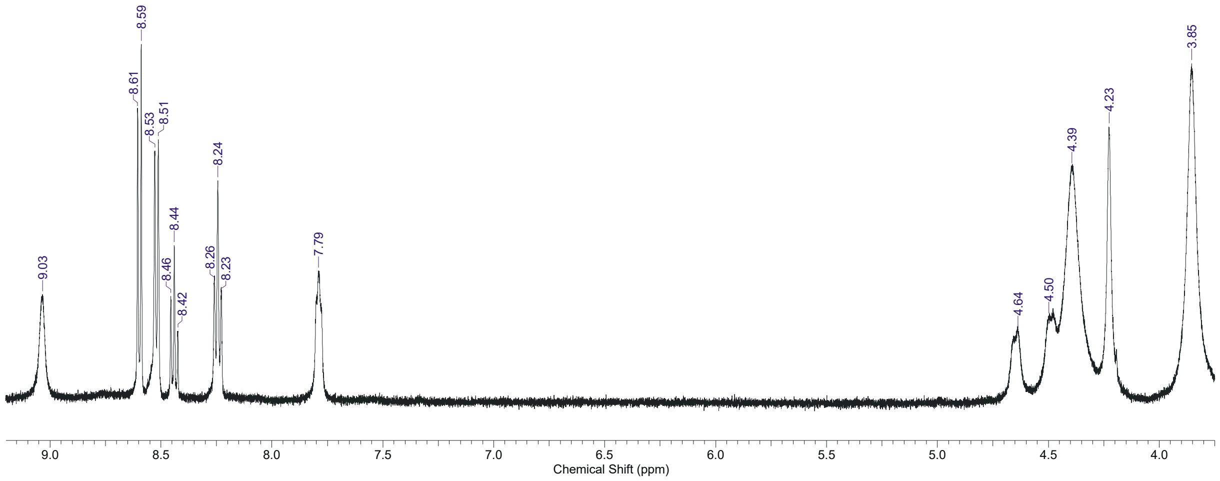

Figure S17. ^{1}H NMR spectrum of **2** (213 K).

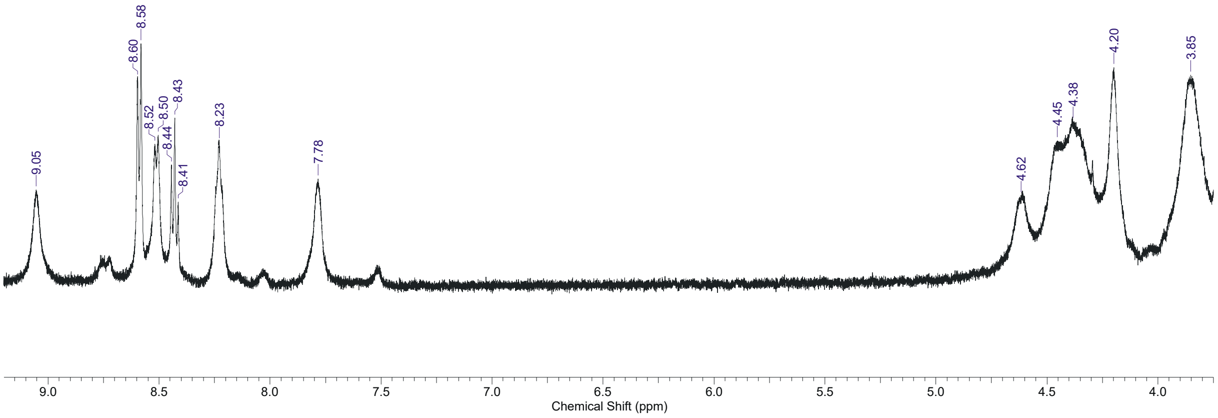

Figure S18. ^{1}H NMR spectrum of **2** (193 K).

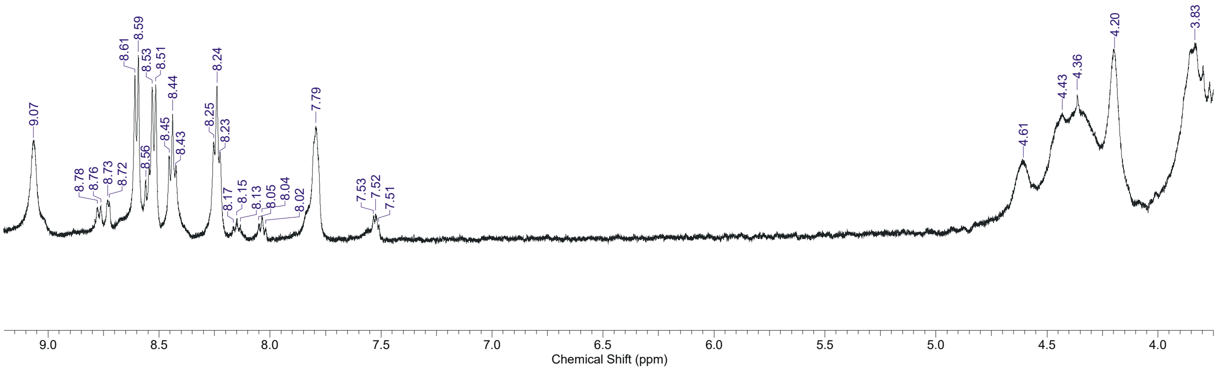

Figure S19. ^{1}H NMR spectrum of **2** (183 K).

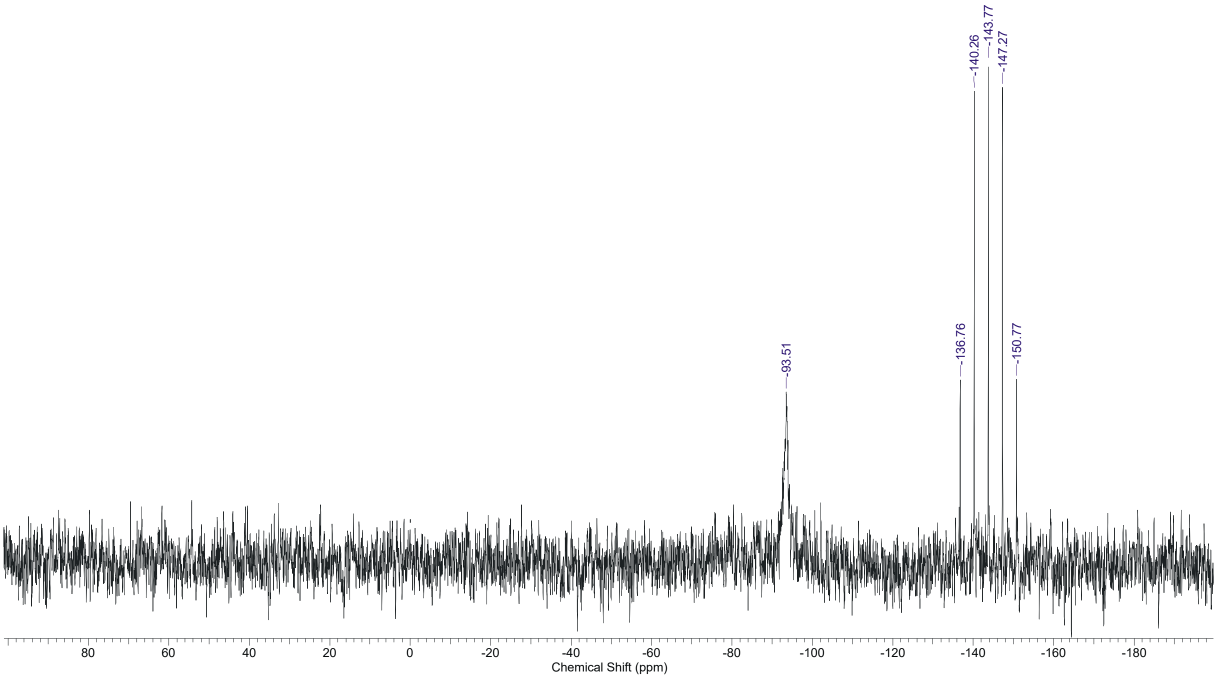

Figure S20. $^{31}P\{^{1}H\}$ NMR spectrum of **2** (183 K).

Table S1. Proposed stacking interaction between aromatic rings.

Cg(I), Cg(J)	Cg-Cg [Å]	Cg(I)_Perp	Alpha
Cg1 – Cg2[i]	5.4825(11)	4.2954(8)	58.06(9)
Cg1 – Cg3[i]	4.5640(12)	3.9505(8)	21.14(10)
Cg2 – Cg1[ii]	5.4826(11)	3.7808(8)	58.06(9)
Cg3 – Cg1[ii]	4.5640(12)	2.8756(8)	21.14(10)

symmetry codes: (i) 0.5-x, 0.5+y, 1.5-z; (ii) 0.5-x, -0.5+y, 1.5-z

Cg(I), Cg(J) – plane numbers; Cg1: ring N31-C32-C33-C34-C35-C36; Cg2: ring N41-C42-C43-C44-C45-C45; Cg3: ring N51-C52-C53-C54-C55-C56

Cg-Cg – distance between ring centroids

Cg(I)_Perp – perpendicular distance of centroid of ring Cg(I) on ring J

Alfa - dihedral angle between planes I and J

Table S2. Analysis of C-H···π interactions**.**

C-H···Cg(I)	H-Cg [Å]	H_Perp [Å]	C···Cg [Å]	C-H···Cg [°]	C-H···π [°]
C12-H12A – Cg3	2.87	2.80	3.740(2)	148	59
C22-H22B – Cg1[i]	2.98	2.96	3.752(2)	136	41

symmetry code: (i) 0.5-x, -0.5+y, 1.5-z

Cg(I) – centroid of the ring; Cg1: ring N31-C32-C33-C34-C35-C36; Cg3: ring N51-C52-C53-C54-C55-C56

H-Cg – distance between hydrogen atom and ring centroid

H_Perp – perpendicular distance of hydrogen atom to ring plane

C-Cg – distance between carbon atom and ring centroid

C-H···Cg – C-H···Cg angle

C-H···π - angle of the C-H bond with the π-plane of the ring

Table S3. Analysis of potential C-H···X hydrogen bonds.

C-H···A	C-H [Å]	H···A [Å]	C···A [Å]	C-H···A [°]
C22-H22B – N51	0.99	2.60	3.296(3)	128
C55-H55 – N23^{i}	0.95	2.55	3.453(3)	159
C21-H21B – F4^{ii}	0.99	2.47	3.363(3)	149
C33-H33 – F3^{iii}	0.95	2.50	3.331(2)	146
C44-H44 – F6^{iv}	0.95	2.52	3.275(3)	137
C25-H25A – F5^{v}	0.99	2.54	3.461(3)	154
C53-H53 – F1	0.95	2.54	3.461(2)	163

symmetry code: (i) 1+x, y, z; (ii) -0.5+x, 1.5-y, 0.5+z; (iii) -1+x, y,z; (iv) 1-x, 1-y, 1-z; (v) 0.5-x, -0.5+y, 1.5-z
